# Rat models of human diseases and related phenotypes: a systematic inventory of the causative genes

**DOI:** 10.1186/s12929-020-00673-8

**Published:** 2020-08-02

**Authors:** Claude Szpirer

**Affiliations:** 1 Université Libre de Bruxelles, B-6041 Gosselies, Belgium; 2 Waterloo, Belgium

**Keywords:** Rat, Disease, Genes, Animal models

## Abstract

The laboratory rat has been used for a long time as the model of choice in several biomedical disciplines. Numerous inbred strains have been isolated, displaying a wide range of phenotypes and providing many models of human traits and diseases. Rat genome mapping and genomics was considerably developed in the last decades. The availability of these resources has stimulated numerous studies aimed at discovering causal disease genes by positional identification. Numerous rat genes have now been identified that underlie monogenic or complex diseases and remarkably, these results have been translated to the human in a significant proportion of cases, leading to the identification of novel human disease susceptibility genes, helping in studying the mechanisms underlying the pathological abnormalities and also suggesting new therapeutic approaches. In addition, reverse genetic tools have been developed. Several genome-editing methods were introduced to generate targeted mutations in genes the function of which could be clarified in this manner [generally these are knockout mutations]. Furthermore, even when the human gene causing a disease had been identified without resorting to a rat model, mutated rat strains (in particular KO strains) were created to analyze the gene function and the disease pathogenesis. Today, over 350 rat genes have been identified as underlying diseases or playing a key role in critical biological processes that are altered in diseases, thereby providing a rich resource of disease models. This article is an update of the progress made in this research and provides the reader with an inventory of these disease genes, a significant number of which have similar effects in rat and humans.

## Background

Why map and identify genes for rat disease phenotypes or related traits? As already pointed out, the laboratory rat (*Rattus norvegicus*) is more than a big mouse. The mouse is a species which has been the mammalian genetic model of choice for a long time, with an initial focus on monogenic traits. Rat models of monogenic traits and diseases have also been isolated but the rat has essentially been a key model for studies of complex traits in fields such as physiology, cardiovascular and diabetes research, arthritis, pharmacology, toxicology, oncology and neurosciences [1–6]. In some situations the rat seems to be a more relevant or faithful model. For instance, the physiology of the rat is extremely well documented, in part because its larger body size affords the opportunity for serial blood draws, which are almost impossible in the mouse; in cardiovascular research [7], sophisticated surgical manipulations, and physiological measurements such as blood pressure measurements by telemetry are easier to perform and more reliable in rats compared to mice [1, 3]. The rat has also long been a common choice for pharmacology and toxicology studies because it shares a similar pathway with humans for eradicating toxins [8]. With respect to cancer research [9, 10], and more precisely mammary cancer research, it is noteworthy that rat and human carcinomas show similar development and histopathological features [11, 12]; furthermore, rat mammary tumors are strongly hormone dependent for both induction and growth, thus resembling human breast tumors and no virus appears to be involved in rat and human mammary carcinogenesis, unlike mouse mammary carcinogenesis the etiological agent of which is the mouse mammary tumor virus. As stated by Russo “The rat mammary tumor model is well suited for studying in situ and invasive lesions […]. The classification of the tumors matches well with the criteria used in the human pathology, and provides an adequate model for understanding these phases of the human disease” [11]. In addition, there is extensive overlap between human breast and rat mammary cancer susceptibility genomic regions and “the laboratory rat will continue to be an important model organism for researching genetically determined mechanisms of mammary cancer susceptibility that may translate directly to human susceptibility” [13]. In neuroscience research, rats have significant anatomical and behavioral advantages over mice, because they are more sociable and skilled and have complex cognitive abilities; this wider range of social behaviors and a richer acoustic communication system confer the rat advantages in comparison to mouse models to study neuro-developmental disorders and in particular autism [14, 15]. The rat thus provides one with particularly reliable models of human traits or diseases [3, 8, 11–17] (multiple details emphasizing the value of rat models can be found in these articles).

Numerous rat strains have been created by selective breeding of animals expressing a desired phenotype, generating a very large collection of genetic models of pathological complex, polygenic traits, most of which are quantitative. Interestingly, these strains also provide one with additional phenotypes, which were not selected for. Just as the traits that were selected for, most of these phenotypes are polygenic. All these phenotypes can be used as models of human traits or diseases [18], implying that the genes underlying these traits or diseases should be identified. Information on rat strains and rat disease models, can be found at the Rat Genome Database (RGD, https://rgd.mcw.edu/) [19].

In order to give the rat the status of a valuable genetic model, and in particular to identify the genes underlying complex traits by forward genetic approaches and to analyze the relevant biological mechanisms, several tools had to be developed. This has been accomplished. Genetic and chromosome maps have been developed; the genomic sequence of dozens of rat strains has been established; a number of resources have been created to provide investigators with access to genetic, genomic, phenotype and disease-relevant data as well as software tools necessary for their research [3, 20]. Thanks to these resources, positional identification of numerous rat genes underlying monogenic or complex diseases and related traits could be achieved. On the other hand, reverse genetic tools have also been developed. Efficient methods to generate mutant rats became available; sperm N-ethyl-N-nitrosourea (ENU) mutagenesis followed by gene-targeted screening methods lead to the isolation of several mutants, including knockout (KO) strains ([21] and references therein). Rat ES were successfully derived and could be used for targeted mutations by homologous recombination; more importantly, several methods not relying on the use of ES cells were introduced to generated targeted mutations (often these are KO mutations), namely gene editing by zinc finger nucleases, by transcription activator-like effector nucleases and finally by the clustered regularly interspaced short palindromic repeat (CRISPR/Cas) system [22]. Transgenic rats can also be generated, including humanized rats carrying large chromosomic fragments (“transchromosomic humanized” rats) [23]. Development of these technologies provides the researcher with all the tools required to take advantage of the unique opportunities offered by the rat as leading model for studies different areas of biomedical research [3, 17]. In this review I made an inventory of the rat genes identified as responsible for monogenic or polygenic diseases and related traits. I took into account the rat genes identified by forward genetic methods as well as those inactivated by ENU-mutagenesis and by targeted mutations, the inactivation of which generated a disease or an abnormal phenotype. This update of the progress made in the identification of rat disease genes shows that a considerable number of conserved genes have similar effects on biological traits in rats and humans, establishing the rat as a valuable model in studies of the genetic basis of human diseases and thus providing one with a useful resource of disease models.

## Materials and methods

The data (causal genes of rat diseases and related traits) were collected by regular and systematic screening of the biomedical literature, PubMed searches (https://www.ncbi.nlm.nih.gov/) and regular Google Scholar alerts based on the keywords “knockout”, “mutation”, “rat” (spread over several months). In addition, relevant data were retrieved from the RGD (“Disease Portals”), with advices from Jennifer R. Smith. Genes identified by forward genetic means (or by direct molecular sequencing) were considered as suggestive, solid or confirmed, respectively, as indicated in each case in Table 1 (one, two or three asterisks), on the basis of the criterions described in the legend to the table; these criterions are based on the standards discussed by Glazier and coworkers [24]. With respect to the induced mutants, they were included provided they were convincingly shown to be specifically altered. The official gene symbols are used in this article and were obtained from the National Center for Biotechnology Information (https://www.ncbi.nlm.nih.gov/), Gene section. In several instances the original publications did not use the official gene symbol; in these cases, the non-official symbol is indicated in parenthesis in the footnote to the table, where the full name of each gene is described. The position of every gene was also obtained from the NCBI.

## Results

The core of this article is a list of the diseases and related traits or phenotypes the causal gene of which was identified in the rat (Table 1). The genes identified by forward genetic methods or, in a few instances, by direct molecular characterization are labeled by asterisks (see legend to table). Also listed are the phenotypes uncovered by reverse genetics methods, either by ENU-mutagenesis followed by selection of the desired mutated gene (these genes are labeled by the symbol ^ENU^), or by targeted gene editing (these genes are labeled by ^T^). Table 1A shows the monogenic traits, and Table 1B the complex traits (in a few cases this distinction is somewhat arbitrary, but in general this is a useful classification). Of note, when a gene was associated with several distinct phenotypes, an entry was created for each phenotype and the gene thus appears several times in the table. When the human homolog gene is known to be causal of the relevant disease or trait, it is also indicated in the table. Furthermore, entries in bold characters indicate that the human gene was found to be causal as a direct translation of the results obtained in the rat.

### Identification of rat disease genes by forward genetic methods

The identification of gene(s) underlying a given phenotype typically starts with the mapping of the trait by linkage analysis (backcrosses, intercrosses). In the case of monogenic traits, this approach is generally sufficient to identify the causative gene (positional identification, as illustrated in Table 1A). Identifying genes controlling complex traits is much more difficult [24, 25]; indeed, linkage analyses of such traits lead to the localization of quantitative trait loci (QTLs), which are too large to allow the identification of the causative gene. Complementary strategies are thus required to narrow down the list of candidate genes, such as the generation of congenic lines or/and the use of integrative genomic approaches (as discussed in [26]). Alternative approaches rely on the use of panels of lines that show a higher level of recombinant events, as a result of crossing parental strains for multiple generations, such as recombinant inbred strains or heterogeneous stocks (as discussed in [27], for a striking harvest of results derived from the study of a heterogeneous stock, see [28]). The first complex-trait gene identified is the *Cd36* gene, which causes insulin resistance, hyperlipidemia and hypertension in the spontaneously hypertensive rat (SHR) [29, 30]. This identification was based on a combined gene expression micro-array and linkage approach and was definitively proven by in vivo complementation, i.e. transgenic expression of normal *Cd36* in the SHR [31]. Last but not least, association was then demonstrated between human *CD36* and insulin resistance [32]. Subsequently, the tools of forward genetic studies as well as gene expression and/or computational analysis (integrative genomics) led to the identification of numerous genes underlying rat polygenic traits or diseases, such as blood pressure, cardiac mass, diabetes, inflammation (in particular arthritis, encephalomyelitis), glomerulonephritis, mammary cancer, neurobehavioral traits, proteinuria. In several instances, the results were translated to the human, as illustrated in Table 1 by bold entries. Interestingly, a recently discovered complex trait gene is a long non-coding RNA, itself contained within the 5′ UTR of the *Rffl* gene (*Rffl-lnc1*); *Rffl-lnc1* shows a 19 bp indel polymorphism which is the precise variation underlying regulation of blood pressure and QT-interval. This work was based on fine and systematic congenic mapping and is the first one to identify quantitative trait nucleotides in a long non-coding RNA [33]. The human homologous region, on chromosome 17, has multiple minor alleles that are associated with shorter QT-intervals and, is some cases, hypertension [34].

Identifying rat disease genes is not only useful to discover the homologous human disease genes but also helps in studying the mechanisms underlying the pathological abnormalities. After all, this is the essence of an animal model. For instance, the study of the genetic basis of stroke in the stroke-prone SHR strain (SHRSP) led to the conclusion that mitochondrial dysfunction contributes to stroke susceptibility and to hypertensive target organ damage (such as vascular damage); this better understanding of the etiology of the disease can open the door to novel therapies, as briefly discussed below [8, 35, 36].

### The importance of rat models in the era of human genetic studies and genome sequencing

The rat is also a useful model to decipher the biological significance of QTLs identified in human genome-wide association studies (GWAS) aimed at understanding the etiology of common human diseases [37, 38]. These studies pint-point human genomic regions controlling a complex trait, and generally contain several genes; the current methods lack the statistical power to pinpoint the human causative gene. Animal model such as the rat provides one with the possibility to knockout or to mutate in more subtle manner each of the rat genes homolog to the human genes contained in a given GWAS locus. In this way, the possible role of each gene can be evaluated. For instance, Flister and co-corkers [39], studying a multigene GWAS locus controlling blood pressure and renal phenotypes (*AGTRAP*-*PLOD1* locus) used gene targeting in a rat model to test each of the genes contained in this locus. In this way these authors could show that several genes impact hypertension and that multiple causative gene variants cosegregate at this locus; several linked genes thus control blood pressure (*Agtrap*, *Clcn6*, *Mthfr*, *Nppa*, *Plod1*). Furthermore, each of the KO rat models so generated can be used to dissect the biological effects of the gene loss of function.

The genetic basis of human diseases is also actively analyzed by whole genome sequencing; such studies have uncovered several genes underlying diseases or related phenotypes [40, 41] and one can thus questioned the importance of genetic analyses in an animal model. As argued and illustrated above, animal models and the rat in particular, remain valuable tools to analyze the biological mechanisms underlying a phenotype. In addition, transgenesis or gene substitution can also be carried out, in which a human allele can be introduced in the relevant KO rat, in order to verify the role of the human mutation. Alternatively, the rat genome can be directly modified to specifically introduce a mutation similar to the one causing the human trait [40, 42]. If the modified rats exhibit defects similar to those observed in the human patients, it can be concluded that the tested human mutation indeed plays a causal role. In addition, similarly to examples mentioned above, such specifically modified rats provide one with models suitable to study the mechanisms responsible for the abnormalities generated by the mutation and also to carry out pharmacological tests and look for possible new therapies [42].

The need of relevant animal models is also illustrated by the fact that even when the human gene causing a disease is known, mutated rat strains (in particular KO strains) were created to analyze the gene function and the disease pathogenesis (see numerous examples of such gene targetings in Table 1). In 2008, Aitman and coworkers [2] reported a list of 21 rat disease genes (that had been identified by positional cloning). Here I updated the list of rat disease genes; this inventory added numerous genes identified (or deliberately mutated) after 2008, thereby evaluating progress made in the input of rat disease models. The total rat gene number listed in Table 1 exceeds 350, illustrating the vigor of the rat biomedical research which led to enrichment of numerous disease models, with the translation to humans of disease gene discoveries in rats.

### Translation of the rat genetic studies into new treatments of human diseases

The identification of a human disease gene has the potential to develop new therapeutic approaches. For instance, the human gene *NCF4* was found to be associated with arthritis as a translation of studies on a rat arthritis model. The gene encodes a component of the NADPH oxidase complex and these studies catalyzed the development of a new therapy for arthritis, based on the use of oxidative-burst inducing substances [43–45] (see Table 1B, Arthritis, *Ncf1* gene). Another interesting example is that of the gene *SHANK3*: mutations in this gene lead to a neurodevelopmental disorder known as Phelan-McDermid syndrome; to date, no pharmaceutical compounds targeting core symptoms of this human disease are available. A *Shank3*-deficient rat model was generated, which showed disabilities similar to those seen in the Phelan-McDermid syndrome and interestingly, the deficits of the mutant rat could be ameliorated by intracerebroventricular oxytocin administration, implying that exogenous oxytocin administration might have therapeutic potential in human patients [42] (see Table 1A, Phelan-McDermid syndrome model). A third example is provided by the study of rat mutated in the *Pde3a* gene, which recapitulates the phenotype of HTNB (Hypertension with brachydactyly) human patients: the functional data suggest that soluble guanyly cyclase activation could be suitable for the treatment of HTNB patients [46] (See Table 1B, Blood pressure section).

**Table 1:** Alphabetical list of diseases and related traits with their causative rat genes and the human homologs

Rat		Human		Comments	References
Phenotype	Causative gene name^a^ Localisation^c^	Phenotype	Ortholog gene name^b^ Localisation^c^		
**A) Monogenic traits**					
Acidosis (pH homeostasis)	*Kcnj16*^T^ 10, 99.33 Mb	Brugada syndrome (arrhythmias)	*KCNJ16* 17q24.3	The SS KO mutant exhibits hyperventilation at rest and decreased arterial pH, showing that *Kcnj16* plays a role in pH regulation, particularly in the ventilatory CO2 chemoreflex; the gene also controls blood pressure: see below, Polygenic traits	[47]
Addiction	*Bdnf*^T^ 3, 100.77 Mb	–	*–*	The heterozygous SD KO mutant exhibits no cocaine-seeking behavior, unlike WT rats	[48]
Addiction	*Cdh13*^T^ 19, 50.85 Mb	Substance abuse, behavioral disorders	*CDH13* 16q23.3	The SS KO mutant shows a stronger responsiveness to cocaine, metamphetamine and saccharin	[49]
Addiction: opioid consumption	*Grm2*^T^ 2q32, 179.58 Mb	–	*–*	The Wistar KO mutant shows higher heroin self-administration and heroin intake as well as reduced sensitivity to cocaine reward; the results suggest that *Grm2* may play an inhibitory role in opioid action; see also below, Polygenic traits, Addiction: alcohol consumption	[50, 51]
Adiposity (fat pads)	Slc22a18** 1, 216.67 Mb	–	*–*	Positional identification revealed a splicing mutation in the SHR/NCrj rat (which shows reduced fat pad weight); in 3 T3-L1 cells, *Slc22a18* KO leads to reduction in lipid accumulation	[52]
Aganglionosis (spotting lethal: *sl*)	Ednrb** 15q22, 88.00 Mb	Hirschsprung disease	*EDNRB* 13q22	Direct analysis of the gene in *sl* rats revealed a deletion; the mutation was then shown to segregate with the phenotype in congenics; phenotype modulated by modifier genes, including *Gdnf*; in the GK strain, the null mutant causes embryonic death; see also below, Polygenic traits, Blood pressure: captopril effects	[53–59]
ALSP	*Csf1r*	ALSP	*CSF1R*	See Macrophage development	[60]
Amelogenesis imperfecta	Sp6** 10q31, 84.96 Mb	Amelogenesis imperfecta	*SP6* 17q21.32	Direct sequencing of the gene revealed an insertional mutation in a mutant SHRSP strain; the mutation was then shown to segregate with the phenotype; partial complementation in *Sp6* transgenic rats; in the human, *SP6* is one the genes causing Amelogenesis imperfecta	[61]
Analbuminemia	Alb** 14p21, 19.18 Mb	Analbuminemia	*ALB* 4q13.3	Direct cloning of the mutant gene revealed a 7 bp deletion at the splicing donor site in intron H of the analbuminemic rat, which does not produce cytoplasmic albumin mRNA	[62]
Anemia (white spotting rat: *Ws/Ws*)	Kit* 14, 35.07 Mb	–	*–*	Direct sequencing of the *Kit* cDNA revealed a 12 bp deletion in the *Ws/Ws* strain, by comparison with the BN and SD sequences; the gene also controls coat color and unilateral renal agenesis: see below	[63]
Anemia (Belgrade rat)	Slc11a2** 7, 142.03 Mb	–	*–*	Positional identification of the gene (from Belgrade rats) which shows a missense mutation, inactivating iron transport	[64]
Angelman syndrome model	*Ube3a*^T^ 1, 116.59 Mb	Angelman syndrome	*UBE3A* 15q11.2	The SD KO mutant shows delayed reflex development, motor deficits in rearing and fine motor skills, aberrant social communication, impaired touchscreen learning and memory, decreased brain volume and altered neuroanatomy	[65]
Ataxia and seizure (groggy rat)	Cacna1a** 19, 25.45 Mb	FHM1, EA2, SCA6	*CACNA1A* 19p13	Positional identification of the gene which shows a missense mutation in the groggy rat, absent in other strains	[66]
Ataxia- telangiectasia	*Atm*^ENU*,* T^ 8q24, 58.02 Mb	Ataxia- telangectiasia	*ATM* 11q22.3	Rats lacking ATM (missense or KO mutation) display paralysis, neuroinflammation and have significant loss of motor neurons and microgliosis in the spinal cord	[67, 68]
Autism spectrum disorders	*Cntnap2*^T^ 4, 74.70 Mb	Epilepsy (CDFE syndrome) and autism spectrum disorders	*CNTNAP2* 7q35-q36.1	The SD KO mutant shows a delayed maturation of auditory processing pathways and striking parallels to disruptions reported in autism spectrum disorders; see also below: Epilepsy	[69]
Autism spectrum disorders	*Fmr1*^T^ Xq37, 154.68 Mb	Autism spectrum disorders	*FMR1* Xq27.3	The SD KO mutant exhibits abnormalities in autism-relevant phenotypes including juvenile play, perseverative behaviors, and sensorimotor gating; see also below, Fragile X syndrome model	[70]
Autism spectrum disorders	*Nlgn3*^T^ X, 71.20 Mb	Autism spectrum disorders	*NLGN3* Xq13.1	The SD KO mutant exhibits abnormalities in autism-relevant phenotypes including juvenile play, perseverative behaviors, sensorimotor gating and sleep disruptions	[70, 71]
Autism spectrum disorders	*Shank2*^T^ 1, 217.15 Mb	Autism spectrum disorders	*SHANK2* 11q13.3-q13.4	The SD KO mutant exhibits social and repetitive impairments, as well as a profound phenotype of hyperactivity and hypermotivation that can be ameliorated through the administration of dopamine receptor 1 or metabotropic glutamate receptor 1 antagonists	[72]
Brain development (*qc*)	Lmx1a** 13, 85.92 Mb	–	*–*	Positional identification of the gene, probably involved in the development of the ventricular system and dorsal migration of neurons	[73]
Cancer	*Brca2*^ENU^ 12p12, 0.50 Mb	Breast, ovarian and other cancers	*BRCA2* 13q13.1	The SD KO mutant is sterile and develops a variety of tumors; surprisingly, the female KO rat does not show any increased incidence of mammary carcinomas	[74]
Cancer	*Msh6*^ENU^ 6, 11.64 Mb	Lynch syndrome (HNPCC)	*MSH6* 2p16	Diverse tumors appear in the homozygous Wistar KO mutant; the tumors exhibit microsatellite instability	[75]
Cancer	*Tp53*^ENU, T^ 10q24, 56.19 Mb	Li-Fraumeni syndrome	*TP53* 17p13.1	The heterozygous KO mutants (F344, Wistar, DAc8, SD) develop lymphomas or different types of sarcomas (more typical of human tumors than those found in *Tp53* mice mutants), depending on the genetic background; *Tp53* also controls Spermatogenesis: see below, Polygenic Traits	[76–79]
Cancer, colon	*Apc*^ENU^ 18p12, 27.01 Mb	Familial colon cancer	*APC* 5q21-q22	Two models are available; the *Pirc* mutant is homozygous lethal while the heterozygous rat develops polyposis and colon cancers, and thus mimics the human *APC*-dependent neoplasia (unlike the *Apc* mutant mice); the KAD mutant is homozygous, viable and shows enhanced susceptibility to colon cancer-inducing agents	[80–82]
**Cancer, multiple endocrine neoplasia-like syndrome X**	Cdkn1b**, **4q43,** **168.69 Mb**	**Multiple endocrine neoplasia type 4**	***CDKN1B*** **12p13.1**	**Positional identification of the gene (encoding p27**^**Kip1**^**), mutated in the MNX (SD**^**we)**^**rat; subsequently, a causative mutation was found in the*CDKN1B*gene of a patient presenting with pituitary and parathyroid tumors; see also below, Obesity and Polygenic traits, Cancer, mammary gland development**	[83, 84]
Cancer, renal carcinoma	Flcn** 10, 46.15 Mb	Birt-Hogg-Dube syndrome	*BHD* 17p11.2	Positional identification of the gene: frameshift mutation in the Nihon rat gene, causing a dominant phenotype; LOH in tumors	[85]
Cancer, renal carcinoma (Eker rat)	Tsc2** 10q12, 13.96 Mb	Renal carcinoma	*TSC2* 16p3.13	Positional identification of the gene; deletion of the 3′ end of the gene; LOH in tumors, which only express the mutant mRNA	[86]
Cardiac inflammation and fibrosis	*Sh2b3*^T^ 12, 40.26 Mb	Increased risk of myocardial infarction	*SH2B3* 12q24	The SS KO mutant shows exacerbated chronic inflammation and fibrosis post myocardial infarction; the gene also controls blood pressure: see below, Polygenic Traits	[87]
Cardiac ischemia	*Il1rl2*^T^ 9, 47.04 Mb	–	*–*	An SD mutant was generated with cardiac-specific *Il1rl2 (Il36r)* KO; this mutant shows improved cardiac function, reduced inflammatory response and apoptosis after ischemia-reperfusion	[88]
Cardiac ischemia	*Ubd*^T^ 20, 1.87 Mb	–	*–*	The SD KO mutant shows cardiac dysfunction and increased cardiomyocyte apoptosis after myocardial infarction, associated with reduced *Cav3* expression; the gene also controls type 1 diabetes (see below)	[89]
Cardiomyopathy	*Dnmt1*^T^ 8, 21.92 Mb	–	*–*	An SD mutant was generated with cardiac-specific *Dnmt1* KO; this mutant shows protection against pathological injury induced by adryamycin (increased expression of *DNMT1* is observed in familial hypertrophic cardiomyopathy patients)	[90]
Cardiomyopathy (hypertrophic)	*Myh7b*^T^ 3, 151.10 Mb	Hypertrophic cardiomyopathy	*MYH7B* 20q11.22	Mutations were found in a small fraction of human patients; the KO rat mutant shows hypertrophic cardiomyopathy and cardiac fibrosis; the CaMK-signaling pathway is overexpressed in the mutant cardiomyocytes	[91]
Cardiomyopathy(atrial)	*Myl4*^T^ 10, 92.63 Mb	Atrial cardiomyopathy	*MYL4* 17q21.32	The KO mutant reproduces the clinical phenotype, showing atrial arrhythmia, left atrial dilation and progressive atrial fibrosis	[40]
Cardiomyopathy	Rbm20** 1, 274.39 Mb	Dilated cardiomyopathy	*RBM20* 10q25.2	Positional identification of the gene; deficiency of *Rbm20* alters splicing of several transcripts, such as titin and reduces exercise capacity	[92]
Cataract (NUC1 rat)	*Cryba1* 10, 65.16 Mb	Cataract	*CRYBA1* 17q11.2	Positional identification of the gene: insertion in exon 6 of the NUC1 rat; the mutation is recessive and impairs the development of the retinal pigmented epithelium	[93, 94]
Cataract	Crygd** 9q32, 71.77 Mb	–	*–*	Positional identification of the gene: mutation in the start codon of the gene in the SS/Jr-Ctr strain	[95]
Cataract	Gja3** 15p12, 41.15 Mb	Cataract	*GJA3* 13q12.11	Positional identification of the gene: non-conservative base substitution in the gene in a SHRSP-derived strain	[96]
Cataract	Gja8** 2, 199.05 Mb	Cataract	*GJA8* 1q21	Positional identification of the gene; 2 rat strains show dominant cataract due to non-conservative base substitutions (SHR-Dca and UPL); the SHR-Dca homozygote exhibits microphthalmia; this mutation also lowers blood pressure; see also below, Polygenic Traits, Blood pressure	[97, 98]
**Cataract**	Lss** **20, 12.84 Mb**	**Cataract**	***LSS*** **21q22.3**	**Positional identification of the gene: abnormal splicing in the Shumiya cataract rat; phenotype modified by*Fdft1*(15, 50.10 Mb); both genes affect cholesterol synthesis; lanosterol treatment reduces cataract severity**	[99, 100]
Cataract (*kfrs4* mutation)	Mip** 7, 2.64 Mb	Cataract	*MIP* 12q13.3	Positional identification of the gene which, in the mutant, shows a 5 bp insertion leading to a frameshift mutation producing a truncated protein	[101]
Chediak-Higashi syndrome model (*beige*)	Lyst* 17, 90.32 Mb	Chediak-Higashi syndrome 1	*LYST* 1q42	Direct sequencing of the mutant rat *beige* gene revealed the presence of a large deletion	[102]
Cerebellar vermis defect (*cvd*)/ Hobble (*hob*)	Unc5c** 2q44, 247.05 Mb	–	*–*	Positional identification of the gene; the rat mutation is homolog to the mouse rostral cerebellar malformation mutation in the gene encoding netrin receptor C	[103]
Coat color: albinism; siamese	Tyr***^,T^ 1q32, 151.01 Mb	Ocolocutaneous albinism	*TYR* 11q14.3	Positional identification of the *siamese* mutant; an albino DA KO mutant was also generated and correction of the albino mutation was done using the CRISP-Cas system	[104–107]
Coat color: nonagouti	Asip*** 3, 150.49 Mb	–	*–*	Cloning of the basis of homology with the mouse variant: deletion in exon 2 of the nonagouti variant; correction of the mutation using the CRISP-Cas system	[107, 108]
Coat color: head spot (KFRS4/Kyo rat)	Ednrb** 15q22, 88.00 Mb	Waardenburg syndrome type 4	*EDNRB* 13q22	Positional identification of the gene; no mutation found in the gene but a deletion was identified upstream the gene in a putative enhancer	[109]
Coat color: hooded (*h*) and the *Ws/Ws rat*	Kit*** 14, 35.07 Mb	–	*–*	Positional identification of the gene: two different insertions found in two alleles (*h* and *h*^*T*^); correction of the hooded mutation using the CRISP-Cas system; the gene is also mutated in the white spotting rat (*Ws/Ws*) (no melanocytes) and seems to control anemia (see above); it is also involved in Unilateral renal agenesis (see below)	[63, 107, 110]
Cockayne syndrome (CS) model	*Ercc6*^T^ 16, 8.73 Mb	Cockayne syndrome	*ERCC6* 10q11.23	The SD KO mutant displays DNA repair-deficient phenotypes and brain abnormalities, features that resemble those of CS patients	[111]
Congenital stationary night blindness	Cacna1f** X, 15,71 Mb	Congenital stationary night blindness	*CACNAIF* Xp11.23	Direct sequencing of the cDNA revealed a mutation generating a stop codon in a strain of spontaneous mutant rat; in a backcross the mutation was found to segregate with the phenotype	[112]
Creeping (*cre*)	Reln** 4q11, 9.35 Mb	Lissencephaly	*RELN* 7q22	Positional identification of the gene, mutated in the KZC rat; the rat mutant is homolog to the mouse *reeler*	[113]
Cystic fibrosis	*Cftr*^T^ 4q21, 42.69 Mb	Cystic fibrosis	*CFTR* 7q31.2	Three mutant strains were described: two KO mutants and a mutant carrying the most frequent human mutation (F508del); they recapitulate many aspects of the human disease (defects in airway mucus production and tracheal development, involution of the vas deferens, intestinal obstruction…..); see also below, Polygenic traits, Bone growth	[114, 115]
Cystic leukoencephalopathy model	*Rnaset2*^T^ 1, 53.17 Mb	Cystic leukoencephalopathy	*RNASET2* 6q27	The SD KO mutant shows no brain cystic lesions but exhibits enlarged prefrontal cortex and hippocampal complex as well as memory deficits (less severe neurodegeneration phenotype than the human patients)	[116]
Cystinosis	Ctns** 10, 59.75 Mb	Cystinosis	*CTNS* 17p13.2	Positional identification of the gene, partially deleted in the Long-Evans Agouti rat; the mutation also causes renal glucosuria	[117]
Danon disease model	*Lamp2*^T^ Xq35, 124.72 Mb	Danon disease	*LAMP2* Xq24	The SD KO rat shows great similarity to human patients: hypercholesterolemia, hyperglycaemia, cardiomyopathy, and other disorders including retinopathy and chronic kidney injury	[118]
D-amino-acid oxidase deficiency	Dao** 12, 48.35 Mb	–	*–*	The LEA rat strain lacks DAO activity; sequencing of the gene revealed a 54.1 Kb deletion in the LEA strain, but not in other strains; a congenic strain carrying the mutant allele in the F344 genetic background was generated: this congenic strain shows defects in D-amino-acids metabolism	[119]
Deafness (*dfk*: deafness Kyoto)	Kncq1** 1q41, 223.15 Mb	Long-QT syndrome, deafness	*KCNQ1* 11p15.5	Positional identification of the gene, partially deleted in the *dfk* rat, which is also hypertensive	[120]
Deafness	Myo7a** 1, 163.00 Mb	Usher syndrome 1B	*MYO7A* 11q13.5	Positional identification of an ENU-induced mutation in the Wistar rat strain (tornado phenotype)	[121]
Deafness; Kyoto circling (*kci*)	Pcdh15** 20, 14.95 Mb	Usher syndrome 1F	*PCDH15* 10q21	Positional identification of the gene, which shows a premature stop codon in the *kci* mutant	[122]
Deafness, retinal dysfunction	Myo15a** 10, 46.84 Mb	Deafness, DFNB3	*MYO15A* 17p11.2	Positional identification of the gene which shows a non-conservative base substitution in the LEW/Ttm-ci2 rat, causing both deafness and blindness	[123]
Demyelination (see also below: Hypomyelination)	*Aspa*^T^ 10, 59.84 Mb	Canavan disease	*ASPA* 17p13.2	The F344 KO mutant shows abnormal myelination in the central nervous system (but no tremor); see also below, Tremor	[124]
Demyelination (*les*)	Mbp* 18, 79.33 Mb	–	*–*	Sequencing of the *les Mbp* gene revealed that it contains a large insertion altering the splicing of the *Mbp* RNA	[125]
Demyelination (*dmy*)	Mrs2*** 17, 42.64 Mb	–	*–*	Positional identification of the gene; complementation by cDNA transgenesis in the *dmy*/*dmy* rat, which carries an inactivating novel splice acceptor site	[126]
Demyelination (*md*)	Plp1** X, 107.50 Mb	–	*–*	The mutation is linked to the X chromosome; sequencing of the mutant *Plp1* cDNA revealed a missense mutation, probably inducing a conformational change in the protein (homologous to the *jimpy* mouse mutant)	[127]
Demyelination (*Taiep*)	Tubb4a** 9, 9.96 Mb	Hypomyelination	*TUBB4A* 19p13.3	Positional identification of the gene which shows a missense mutation in the *Taiep* rat	[128]
Diabetes insipidus	Avp*** 3q35, 123.12 Mb	Neurohypophys-eal diabetes insipidus	*AVP* 20p13	Direct cloning of the gene which shows a single base deletion in the Brattleboro rat; complementation by transgenesis in the hypothalamus	[129, 130]
Diabetes insipidus	Stim1** 1, 167.37 Mb	Autoimmunity	*STIM1* 11p15.4	The SHR-A3 strain exhibits nephrogenic diabetes insipidus; genome sequencing revealed a deletion affecting the *Stim1* gene is in the SHR-A3 strain, but not in other strains; the phenotype segregates with the mutation, which compromises store-operated calcium entry; this pleiotropic genes also controls Behavior (stress response), Renal injury and Stroke (see Polygenic traits below)	[131]
Dilute-opisthotonus (*dop*)	Myo5a** 8, 82.04 Mb	Griscelli syndrome type I	*MYO5A* 15q21.2	Direct sequencing of the cDNA revealed an in frame, 47aa deletion in the *dop Myo5a* gene, leading to under-expression of the protein (resulting in diluted coat color and ataxia); a second mutant was identified later by whole genome sequencing: it shows several pleiotropic neuropathological and biochemical alterations leading to neurodegeneration	[132, 133]
Duchenne muscular dystrophy	*Dmd*^T^ Xq22, 51.15 Mb	Duchenne muscular dystrophy	DMD Xp21.2-p21.1	Wistar or SD KO rats show several muscle abnormalities (necrosis, fibrosis, reduced strength, reduced motor activity) and dilated cardiomyopathy	[134, 135]
Drug behavioral effects	*Ghsr*^ENU^ 2, 113.06 Mb	–	–	Cocaine-treated FHH mutant rats shows diminished development of cocaine locomotor sensitization relative to WT rats; see also below, Food intake	[136]
Drug metabolism	*Abcb1a*^T^ 4q12, 22.34 Mb	–	–	Wistar or SD KO mutants show increased brain penetration of drugs and other alterations in drug pharmacokinetic parameters	[137–140]
Drug metabolism	*Abcg2*^T^ 4, 88.76 Mb	–	–	The SD KO mutant shows increased brain penetration of drugs and other alterations in drug pharmacokinetic parameters; see also below, Hyperbilirubinemia	[138, 139]
Drug metabolism	*Cyp2c11*^T^ 1q53, 257.68 Mb	–	*–*	The SD KO mutant male shows reduced fertility (CYP2C11 is a male-specific cytochrome P450); expression of other P450’s is upregulated; in vivo, no significant differences were found in drug metabolism	[141]
Drug metabolism	*Cyp2e1*^T^ 1q41, 213.51 Mb	–	*–*	The SD KO rat is physiologically normal, shows a compensatory expression of CYP3A1 and impaired metabolism of chlorzoxazone, a CYP2E1 substrate	[142]
Drug metabolism	*Cyp3a1*^T^ 12, 110.539 Mb *+ Cyp3a2*^T^ 12, 116,41 Mb	–	*–*	The double SD KO rat is physiologically normal but shows increased testosterone serum concentrations; it also shows a compensatory expression of several cytochrome isoforms and impaired metabolism towards CYP3A1/2 substrates	[143]
Dwarfism (Spontaneous dwarf rat)	Gh** 10q32, 94.48 Mb	Dwarfism	*GH* 17q24	Direct cloning of the gene revealed a point mutation causing abnormal splicing in the Spontaneous dwarf rat	[144]
Dwarfism (*mri*)	Prkg2** 14, 12.22 Mb	Growth retardation	*Candidate: PRKG2* 4q13.1-q21.1	Positional identification of the gene; complementation in cultured chondrocyte by cDNA transfection (restoration of differentiation)	[145–147]
Dwarfism (*rdw* rat)	Tg** 7, 107.47 Mb	–	*–*	Sequencing of the *Tg* cDNA from the *rdw* rat revealed a missense mutation; rescue from dwarfism was obtained by thyroid function compensation in *rdw* rats	[148, 149]
Dwarfism (*lde/lde* rat)	Wwox**			See below, Epilepsy	
Dystonia type 25	*Gnal*^T^ 18q12, 62.80 Mb	Dystonia type 25	*GNAL* 18p11	The SD KO mutant shows early-onset phenotypes associated with impaired dopamine transmission, such as reduction in locomotor activity and an abnormal motor skill learning ability; it may be a valuable tool for finding a suitable treatment for dystonia type 25	[150]
Ear and eye development (*dumbo* mutation)	Hmx1** 14, 80.54 Mb	Oculo-auricular syndrome	*HMX1* 4p16.1	Positional identification of the gene; large deletion, 80 kb downstream the *dumbo* rat gene, which is not expressed in the embryo craniofacial mesenchyme	[151]
Eosinophilia (MES rat)	Cyba*** 19, 55.25 Mb	–	*–*	Positional identification of the gene; the mutant gene is deleted in the 5′ splice site of intron 4, leading to an abnormal mRNA and absence of NADPH oxidase activity; the normal phenotype was restored by transgenesis of the normal gene	[152]
Epilepsy (*flathead* rat)	Cit** 12, 46.33 Mb	Microcephaly	*CIT* 12q23.24	Positional identification of the gene, which shows a single base deletion in the mutant rat (*fh*/*fh*), generating a stop codon; cytokinesis is defective in neuronal progenitors; this mutation also leads to microcephaly (see below)	[153, 154]
Epilepsy	*Cntnap2*^T^ 4, 74.70 Mb	Epilepsy (CDFE syndrome) and autism spectrum disorders	*CNTNAP2* 7q35-q36.1	An SD KO mutant exhibits motor seizures, hyperactivity and increased consolidation of wakefulness and rapid eye movement sleep; see also above: Autism spectrum disorders	[155]
Epilepsy (and ataxia)	*Kcna1*^ENU^ 4q42, 159.19 Mb	Episodic ataxia type 1	*KCNA1* 12p13.32	An F344 ENU-induced mutant showing dominant myokimia, neuromyotonia and epileptic seizures was used for positional identification of the gene; expression studies in Xenopus oocytes	[156]
Epilepsy (ADLTE mutant)	*Lgi1*^ENU^ 1, 256.95 Mb	Epilepsy (ADLTE)	*LGI1* 10q23.33	The F344 mutant shows early-onset spontaneous epileptic seizures and audiogenic seizure susceptibility; astrocytic *Kcnj10* expression is down-regulated	[157, 158]
Epilepsy (febrile seizure; *Hiss* rat)	*Scn1a*^ENU^ 3, 52.39 Mb	Febrile seizure, epilepsy	*SCN1A* 2q24.3	The *Hiss* mutant shows impaired GABA receptor-mediated synaptic transmission	[159]
Epilepsy	*Sv2a*^ENU^ 2, 198.32 Mb	Epilepsy, microcephaly	*SV2A* 1q21.2	The F344 mutant shows a high susceptibility to the development of kindling	[160]
Epilepsy (*lde*/*lde* rat)	Wwox** 19, 46.76 Mb	–	*–*	Positional identification of the gene, mutated (13 bp deletion) in the *lde*/*lde* rat which also shows dwarfism and abnormal testis development (these phenotypes are simultaneously inherited as a single trait)	[161]
Fabry disease model	*Gla*^T^ X, 105.41 Mb	Fabry disease	*GLA* Xq22.1	The DA KO mutant manifests symptoms similar to those seen in Fabry patients such as altered touch and pain detection; the sensory neuron cell membrane is sensitized to mechanical probing	[162]
Food intake	*Ghsr*^ENU, T^ 2, 113.06 Mb	–	*–*	The FHH mutant shows reduced intake of palatable, high-calorie food (see also above, Drug behavioral effects); the Wistar KO rat shows reduced body weight and blunted food consumption	[163–165]
Fragile X syndrome model	*Fmr1*^T^ Xq37, 154.68 Mb	Fragile X syndrome	*FMR1* Xq27.3	Two SD KO strains are available; they show disrupted cortical processing of auditory stimuli, hippocampal cellular and synaptic deficits, memory defects, abnormal visual responses, impaired spatial learning, attention deficits (deletion of the KH1 domain); see also above, Autism spectrum disorders	([166, 167] and references therein, [168])
Fused pulmonary lobes (*fpl*)	Frem2** 2, 142.75 Mb	Fraser syndrome	*FREM2* 13q13.3	Direct sequencing of the *fpl* cDNA showed a premature stop codon; similarity with the mouse *Frem2* mutant	[169]
Glycogenosis (PHK deficiency; *gsd* rat)	Phkg2** 1, 199.02 Mb	Glycogenosis	*PHKG2* 16p11.2	Direct sequencing of the human and rat cDNA’s revealed mutations in patients and in the *gsd* rat	[170]
Hairlessness	Hr** 15, 52.24 Mb	Alopecia, atrichia	*HR* 8p21.2	ENU-induced mutant (Kyoto rhino rat) selected on the basis of the phenotype and then positional identification of the gene; the mutant shows hair loss as well as proteinuria and glomerulosclerosis	[171]
Hairlessness	Krt@** 7q36, ~ 141 Mb	–	–	Positional identification of the locus revealing a 80 kb deletion of several keratin genes in the Hirosaki hairless rats	[172]
Hairlessness (*rex* mutation)	Krt71** 7q36, 143.35 Mb	–	–	Positional identification of the gene which has a 7 bp deletion at the splicing acceptor site of the *rex* intron 1; curly hair in heterozygotes; hair loss in homozygous	[173]
Hairlessness	Prss8** 1, 199.37 Mb	–	–	Positional identification of the gene: mutations found in affected rats (CR hairless and fuzzy)	[174, 175]
Hairlessness and dermatitis	Trpv3** 10, 59.83 Mb	–	*–*	Direct sequencing of the rat cDNA, after positional identification of the mouse gene: dominant, missense mutation in the WBN/Kob-Ht rat and the DS-Nh mouse	[176]
Hemochromatosis	Tfr2* 12q12, 22.18 Mb	Hemochromatosis	*TFR2* 7q22	Direct sequencing of the gene revealed an Ala679Gly polymorphism; homozygosity for this SNP is associated with the mutant phenotype in a Hsd:HHCL Wistar stock	[177]
Hemophilia A (*WAG-F8m1Ycb*)	F8**^,T^ 18, 367.17 Mb	Hemophilia A, hemophilic arthropathy	F8 Xq28	Evaluation of the individual clotting factors revealed a missense mutation in the factor FVIII cDNA of the mutant rat; the hemostatic defect was corrected by administration of human factor VIII; two KO mutants show an hemophilic phenotype and seems to be good models of hemophilic arthropathy or bone transplantation	[178–181]
Heriditary tyrosinemia type I model	*Fah*^T^ 1, 146.71 Mb	Heriditary tyrosinemia type I	*FAH* 15q25.1	The SD KO mutant shows the major manifestations of the human disease: hypertyrosinemia, renal tubular damage and liver fibrosis and cirrhosis; Cas9n-mediated genome editing was used to correct the defect	[182, 183]
HPS model: Ruby/Red eye dilution (platelet storage disease)	Rab38* 1, 152.07 Mb	HPS	–	Direct sequencing of the gene; same mutation in FH and TM rats, probably derived from a common ancestor; lung surfactant secretion is altered in the mutant rats; *Rab38* also controls proteinuria (QTL *Rf2;* see below)	[184, 185]
Hydrocephalus	*Ccdc39*^T^ 2, 120.28 Mb	–	–	The SD KO mutant shows severe hydrocephalus with subarachnoid haemorrhage and inflammatory cell invasion into the perivascular space, as well as impaired glymphatic cerebrospinal fluid flow	[186]
Hydrocephalus	*Ccdc85c*^T^ 6, 132.11 Mb	–	–	The F344 KO mutant shows non-obstructive hydrocephalus, subcortical heterotopia and intracranial hemorrhage	[187]
Hydrocephalus, X-linked	*L1cam*^T^ Xq37, 156.90 Mb	X-linked hydrocephalus	*L1CAM* Xq28	The SD KO male mutant shows reductions in fractional anisotropy and axial diffusivity in the corpus callosum, external capsule, and internal capsule	[188]
**Hyperbilirubinemia**	Abcc2**^,T^ **1, 263.55 Mb**	**Hyperbilirubinemia II / DJS**	***ABCC2*** **10q24**	**Direct sequencing of the cDNA in the Eisai hyperbilirubinemic rat (EHBR) revealed a premature stop codon; the same approach in the TR rat showed a 1 bp deletion; alterations were found in drug pharmacokinetics in an SD KO mutant; mutations were then discovered in the*ABCC2*gene of DJS patients**	[138, 189–191]
Hyperbilirubinemia	*Slco1b2*^T^ 4, 175.81 Mb	Hyperbilirubinemia (Rotor type)	*SLCO1B3* 12p12.2	The SD KO mutant shows increased levels of serum bilirubin and altered pharmacokinetic behavior of pravastatin, an SLCO1B2 substrate; it could be a good model of the human Rotor syndrome	[192]
Hyperbilirubinemia	Ugt1a1*** 9q35, 95.30 Mb	Hyperbilirubinemia, Crigler-Najjar syndrome	*UGT1A* 2q37.1	Direct sequencing of cDNA showed that the Gunn rat has a frameshift mutation in the 3′ region of the gene; correction of the defect could be achieved with recombinant *UGT1A* adenoviruses	[193, 194]
Hypercholesterolemia	*Apoe*^T^ 1, 80.61 Mb	Familial APOE deficiency	*APOE* 19q13.32	An SD KO mutant displays hypercholesterolemia, atherosclerosis, hepatic steatosis and decreased HDL-cholesterol levels; another mutant also shows adventitial immune infiltrates; an *Apoe*/*Ldlr* double KO mutant was also studied by Zhao et al. (2018) [195]	[195–197]
Hypercholesterol-emia	*Ldlr*^ENU, T^ 8, 22.75 Mb	Familial hypercholesterolemia	*LDLR* 19p13.2	The F344 and SD mutants display hypercholesterolemia, hypertriglyceridemia, atherosclerosis, xanthomatosis; hepatic steatosis was also found in the SD mutant	[195, 198, 199]
Hypercholesterol-emia (diet-induced: ExHc rat)	Ppp4r3b** 14, 113.57 Mb	–	*–*	Positional identification of the gene, coupled with gene expression analyses; the gene is under-expressed in the ExHC rat and carries a strain-specific10 bp deletion leading to a premature stop codon	[200]
Hyperuricemia	*Uox*^T^ 2, 252.445 MB	–	No functional gene	The SD KO mutant is viable (unlike the mouse KO) and shows elevated levels of serum uric acid, providing a good model for studying hyperuricemia and associated disorders	[201]
Hypodactyly (*hd*)	Cntrob** 10q24, 55.90 Mb	–	–	Positional identification of the gene; the *hd* allele carries a retroviral insertion; centrobin thus controls both limb development and spermatogenesis	[202]
Hypohidrotic ectodermal dysplasia (*swh*)	Edaradd** 17, 90.80 Mb	Hypohidrotic ectodermal dysplasia	EDARADD 1q42.3	Positional identification of the gene, which shows a missense mutation in the sparse-and-wavy rat (*swh*); sparse hair and oligodontia in this mutant rat and in human patients	[203]
Hypomyelination	*Bace1*^T^ 8, 50.14 Mb	–	*–*	The SD KO mutant shows increased axon density and relatively thinner myelin sheaths around axons of the sciatic nerves; it also shows increased mortality	[204]
Hypothyroidism	*Tshr*^T^ 6q31.2, 115.17 Mb	Congenital hypothyroidism	*TSHR* 14q31.1	The SD KO mutant is infertile and shows the dwarf phenotype as well as suppression of the thyroid-specific genes; the phenotype can be reversed by levothyroxine	[205]
Hypotrichosis (hairlessness)	Dsg4** 18, 12.06 Mb	Hypotrichosis 18q12.1	*DSG4* 18q12	Direct sequencing of the IC hairless rat gene, which shows a large deletion; same approach in the lanceolate hair (*lah*) rat revealed a missense mutation; positional identification of the mutant gene from an SHR congenic strain, which shows a premature termination codon	[206–208]
Immunodeficiency	*Igh*^T^ 6q32, ~ 150 Mb	–	*–*	Two SD KO mutants show absence of Ig and B cells; transgenesis of human *IG* loci reconstitutes B cell development and leads to humanized Ig production	[209, 210]
Immunodeficiency (athymia: *nude*)	Foxn1**, ^T^ 10, 65.62 Mb	Lack of thymus, anencephaly	*FOXN1* 17q11.2	Following positional identification of the mouse gene, the homolog rat gene was found to be mutated in the *nude* strain, disrupting thymus development and hair growth; two induced Wistar mutants were generated: they show thymus deficiency and incomplete hairless	[211–213]
Immuno-deficiency	*Prkdc*^T^ 11q23, 89.29 Mb	Immuno-deficiency, granuloma, autoimmunity	*PRDKC* 8q11.21	The F344 KO mutant shows severe combined immunodeficiency and growth retardation; this mutant was used to establish a model for preclinical testing of human neural precursor cells transplantation as a treatment of neonatal brain damages; a double KO mutant (*Prkdc*^−/−^ and *Il2rg*^−/−^) was also generated; this double mutant shows abolishment of natural killer cells	[214, 215]
Immunodeficiency (SCID)	*Rag1*^T^ 3, 91.21 Mb	SCID	*RAG1* 11p12	The LEW KO mutant shows lymphocyte depletion (and attenuation of hypertension and renal damage: see below, Polygenic traits, Blood pressure)	[216]
Immunodifficiency (SCID)	*Rag2*^T^ 3, 91.19 Mb	SCID	*RAG2* 11p12	The SD KO rat lacks mature B and T cells and was shown to be a viable host for a range of xenograft studies	[217]
Immunodeficiency (SCID)	*Rag1*^T^ 3, 91.21 Mb *Rag2*^T^ 3, 91.19 Mb *Il2rg*^T^ X, 71.17 Mb	–	*–*	The SD triple KO mutant shows impaired development of lymphoid organs, is severely immunodeficient with an absence of mature T, B, and NK cells and supports fast growth of patient-derived xenografts thus holding great potential to serve as a new model for oncology research	[218]
Immunodeficiency (X-SCID)	*Il2rg*^T^ X, 71.17 Mb	X-SCID	*IL2RG* Xq13.1	Two KO mutants are available; they show severe combined immunodeficiency (absence of B and T lymphocytes and of NK cells); a double KO, deficient for both *Il2rg* and *Rag1*, was also described: see above	[219, 220]
Infertility (and cryptorchidism)	*Adamts16*^T^ 1, 36.47 Mb	–	*–*	The KO SS homozygous mutant exhibits cryptorchidism and is infertile; the gene also controls blood pressure (see below, Polygenic traits, Blood pressure)	[221]
Infertility (testicular feminization)	Ar* Xq22, 67.66 Mb	Testicular feminization	*AR* Xq12	Direct sequencing of the gene in a testicular feminized strain: a missense mutation was found in the steroid-binding domain of the androgen receptor	[222]
Infertility	*Bscl2*^ENU^ 1, 225.04 Mb	Congenital generalized lipodystrophy	*BSCL2* 11q12.3	The male KO mutant is infertile and shows small testis and azoospermia (the female is fertile); the gene could be involved in male human fertility; see also below, Monogenic traits, Lipodystrophy and Polygenic traits, Brain development	[223]
Infertility	*Defb23*^T^ 3, 147.93 Mb *Defb26*^T^ 3, 147.98 Mb *Defb42*^T^ 15, 46.16 Mb	–	*–*	The male SD mutant with CRISPR/Cas9-mediated single *Defb* gene disruption has no obvious fertility phenotype but the multiple KO mutant (*Defb23*/*26* or *Defb23*/*26*/*42*) is subfertile	[224]
Infertility (male pseudohermaphrodism: TF rat)	Dhh** 7, 140.58 Mb	Gonadal dysgenesis	*DHH* 12q13.12	Positional identification of the gene which shows a missense mutation in the TF rat; the mutation causes agenesis of Leydig cells and androgen deficiency	[225]
Infertility	*Esr1*^T^ 1q12, 41.19 Mb	–	*–*	Male and female SD KO rats are infertile and show gonadal pathologies; see also below, Polygenic traits, Metabolism	[226]
Infertility	*Esr2*^T^ 6q24.2, 99.16 Mb	–	*–*	Two SD KO mutants were generated; male mutants are fertile while female mutants are infertile (no ovulation); however male mutants exhibit prostatic glandular hyperplasia and changes in expression of genes involved in epithelial proliferation and benign tumor formation; in the female mutants, numerous granulosa cell genes are differentially expressed (including *Kiss1*)	[227–229]
Infertility	*Kiss1*^T^ 13, 50.53 Mb	–	*–*	The KO mutant (male and female) fails to show secretion of luteinising hormone and onset of puberty	[230]
Infertility	*Prdm14*^T^ 5, 5.51 Mb	–	*–*	The WI KO mutant (male and female) fails to generate primordial germ cells and consequently, is infertile	[231]
Infertility (*ifm* mutation)	Sbf1** 7, 130.26 Mb	Charcot-Marie-Tooth disease type 4B3	*SBF1* 22q13.33	Positional identification of the gene, which shows a mutation at a splice site in the *ifm* mutant; homozygous males are infertile (azoospermia); females are normal	[232]
Infertility (tremor rat: TRM/Kyo, carrying the *tm* mutation)	Spata22*** 10q24, 59.89 Mb	–	*–*	Positional identification of a deletion spanning > 200 kb; the *tm* deletion causes infertility and absence-like seizure in both sexes; male infertility was complemented by *Spata22* transgenesis	[233]
Kininogen deficiency (BN/Ka rat)	Kng2** 11, 81.51 Mb	–	*–*	Direct sequencing of the cDNA from the BN/Ka rat revealed a missense mutation which impairs hepatic secretion of the protein; this defect may also render vascular tissue prone to aortic aneurysm formation	[234, 235]
Lipodystrophy, congenital generalized	*Bscl2*^ENU^ 1, 225.04 Mb	Congenital generalized lipodystrophy	*BSCL2* 11q12.3	The KO mutant develops generalized lipodystrophy (lack of white adipose tissue), is glucose intolerant and shows elevated plasma triglyceride and concentrations; see also above: Infertility and below: Polygenic Traits, Brain development	[223]
Lipodystrophy, neuropathy	Lpin1** 6, 41.80 Mb	Rhabdomyolysis Myoglobinuria Metabolic disease traits	*LPIN1* 2p25.1	ENU-induced mutant isolated on the basis of the phenotype and positional identification of the gene; the murine gene is mutated in the *fld* mouse (showing adipocyte defects and demyelination)	[236]
Lymphopenia (T-cell) & IBD	Themis** 1, 17.28 Mb	–	*–*	Positional identification of the gene, which shows a mutation in the BN^m^ rat, impairing *Treg* function	[237]
Microcephaly (*flathead* rat)	Cit** 12, 46.33 Mb	Microcephaly	*CIT* 12q23.24	Positional identification of the gene, which shows a single base deletion in the mutant rat (*fh*/*fh*), generating a stop codon; cytokinesis is defective in neuronal progenitors; this mutation also leads to epilepsy (see above)	[153, 238]
Morphogenesis	*Lpar1*^ENU^ 5, 75.56 Mb	–	*–*	The *Msh6* mutant shows craniofacial disorder and small size	[239]
mTORopathy	*Depdc5*^T^ 14, 83.09 Mb	Epilepsy	*DEPDC5* 22q12.2-q12.3	The homozygous F344 KO rat dies in utero; the heterozygous KO rat displays cortical cytomegalic dysmorphic neurons and has altered cortical neuron excitability (upregulation of the mTORC1 pathway)	[240]
Mucopolysacchar-idosis VI	Arsb*** 2, 23.39 Mb	Mucopolysaccharidosis VI	*ARSB* 5q11-q13	Direct sequencing of the *Arsb* cDNA showed a frame shift mutation with premature stop codon in affected rats (MPR); enzyme replacement therapy	[241, 242]
Multiple mitochondrial dysfunctions syndrome	*Isca1*^T^ 17, 5.28 Mb	Multiple mitochondrial dysfunctions syndrome	*ISCA1*	The heterozygous SD KO mutant is normal but the homozygous mutant shows abnormal development at 8.5 days and dies at embryonic stage	[243]
Myogenic response	*Dusp5*^T^ 1, 274.25 Mb	–	*–*	The FHH.1^BN^ congenic KO mutant shows greater myogenic response of cerebral arteries and enhanced autoregulation of cerebral blood flow	[244]
Neurological disorder (*frogleg* mutation)	Bckdk** 1, 199.35 Mb	Autism and epilepsy	*BCKDK* 16p11.2	Positional identification of the gene which shows a critical missense mutation in the *frogleg* rat, causing abnormalities in hind limb function, reduced brain weight, infertility, seizures	[245]
Neuropathy (Chemotherapy-induced peripheral neuropathy)	*C3*^T^ 9, 9.72 Mb	–	–	C3 is activated by neuronal cells in WT rats after paclitaxel administration; the KO mutant rat has reduced intradermal nerve fiber loss and mechanical allodynia after paclitaxel treatment	[246]
NGLY1-deficicency model	*Ngly1*^T^ 15, 10.40 Mb	NGLY1-deficiency	*NGLY1* 3p24.2	The SD KO mutant shows developmental delay, movement disorder, somatosensory impairment, axonal degradation in the sciatic nerves and scoliosis, in agreement with the symptoms of human patients	[247]
Obesity	Cdkn1b* 4, 168.69 Mb	Multiple endocrine neoplasia type 4	*CDKN1B* 12p13.1	The MNX (SD^we^) rat is mutated in the *Cdkn1b* gene and shows multiple endocrine neoplasia syndrome; this mutant produces elevated levels of ghrelin and shows increased food intake with enhanced body fat mass; see also above, Cancer, Multiple endocrine neoplasia-like syndrome X and below, Polygenic traits, Cancer, Mammary gland development	[248]
Obesity	*Lep*^T^ 4, 56.34 Mb	Obesity	*LEP* 7q31	Targeted and ENU-induced mutations; F344 and SD KO rats are obese, infertile and immunodepressed	[249, 250]
Obesity	Lepr**^,T^ 5*,* 120.50 Mb	Obesity	*LEPR* 1p31	Positional identification of the gene; missense or stop mutation in the Zucker *fa* and Koletsky *obese* (“corpulent”) rats, respectively; the SD KO mutant confirms the phenotype of the spontaneous mutant, with glucose intolerance, hyperinsulinemia, dyslipidemia and diabetes complications	[251–253]
Obesity	*Mc4r*^ENU^ 18, 62.61 Mb	Obesity	*MC4R* 18q22	The MSH6 KO mutant shows increased food intake and adipose mass	[254]
Osteochondrodysplasia (*ocd*)	Golgb1** 11, 66.76 Mb	–	*–*	Positional identification of the gene; the mutant shows an abnormal skeletal system and systemic edema	[255]
**Osteopetrosis (incisors absent:*ia*)**	Plekhm1** **10, 91.45 Mb**	**Osteopetrosis**	***PLEKHM1*** **17q21.31**	**Positional identification of the gene: frameshift mutation in the*ia*rat; mutations discovered in the*PLEKHM1*gene of osteopetrosis patients**	[256]
Osteoporosis pseudoglioma model	*Lrp5*^T^ 1, 218.82 Mb	Osteoporosis pseudoglioma	*LRP5* 11q13.2	Three independent SD KO lines were generated: they display decreased trabecular bone mass and quality as well as sparse and disorganized superficial retinal vasculature as seen in *LRP5-*deficient humans	[257]
Parkinson disease model	*Lrrk2*^T^ 7, 132.86 Mb	Familial PD (dominant)	*LRRK2* 12q12	The Long Evans KO mutant displays weight gain and an abnormal kidney, lung and liver phenotype	[258, 259]
Parkinson disease model	*Nr4a1*^ENU^ 7, 142.90 Mb	–	*–*	The FHH KO mutant shows reduced dopamine cell loss and dyskinesia in this experimental Parkinson disease model; the gene also controls renal function and stress: see below, Polygenic traits, Renal injury and Behavior, stress response	[260]
Parkinson disease model	*Park7*^T^ 5, 167.98 Mb	Familial PD (recessive)	*PARK7* 1p36.23	The Long Evans KO mutant shows motor deficit and age-dependent neuronal loss; *Park7* is also involved in the control of PAH: see below, Polygenic traits, Blood pressure	[261, 262]
Parkinson disease model	*Prkn*^T^ 1, 48.88 Mb	Familial PD (recessive)	*PRKN* 6q26	The Long Evans KO mutant is not different from WT rats	[262]
Parkinson disease model	*Pink1*^T^ 5, 156.68 Mb	Familial PD (recessive)	*PINK1* 1p36	The Long Evans KO mutant shows motor deficit and age-dependent loss of nigral dopaminergic neuronal	[261–263]
Parkinson disease model	Snca* 4, 90.78 Mb	Familial PD (dominant)	*SNCA* 4q22.1	Direct sequencing revealed a mutation in the *Snca* mRNA 3’UTR in a mutant rat, which overexpresses synuclein alpha and shows functional alterations in the dopaminergic and glutamatergic systems	[264, 265]
Phelan-McDermid syndrome model	*Shank3*^T^ 7, 130.47 Mb	Phelan-McDermid syndrome	*SHANK3* 22q13.33	The human neurobehavioral manifestations are due to mutations in *SHANK3*; one of these mutations (a deletion) was introduced in rats, which exhibited disabilities related to those seen in the human patients; these deficits were attenuated by oxytocin treatment	[42]
Pinked eyed dilution (*p*)	Oca2** 1, 114.66 Mb	Oculocutaneous albinism	*OCA2* 15q	Direct sequencing of the *Oca2* cDNA revealed a deletion shared by several mutant strains, that also exhibit the same haplotype, distinct from control strains	[266]
Polycystic kidney disease (ADPKD) (cy/+ rat)	Anks6*** 5, 62.64 Mb	Cystic kidney disease (Nephronophthisis)	*ANKS6*	Positional identification of the gene, mutated in the Han SD (cy/+) rat; overexpression of the mutated variant causes polycystic kidney disease; mutations later found in the human gene	[267–269]
Polycystic kidney disease (ARPKD): nephronophtisis	Nek8** 10, 65.40 Mb	–	*–*	Positional identification of the gene, mutated in the Lewis Polycystic Kidney (LPK) rat, leading to abnormally long cilia on kidney epithelial cells	[270]
Polycystic kidney disease (ARPKD)	*P2rx7*^T^ 12, 39.35 Mb	–	*–*	A *P2rx7* KO was generated in the polycystic kidney (PCK) rat, a model of ARPKD; the mutant shows slower cyst growth and reduction of renal pannexin-1 protein expression and daily urinary ATP excretion	[271]
**Polycystic kidney disease (ARPKD)**	Pkhd1** **9, 26.16 Mb**	**ARPKD**	***PKHD1*** **6p12.2**	**Positional identification of the polycystic kidney (PCK) rat gene, which led to the identification of mutations in the homolog human gene, responsible for ARPKD**	[272]
**Polycystic kidney disease (Wpk rat)**	Tmem67** **5, 27.67 Mb**	**Meckel-Gruber syndrome (MKS3)**	***TMEM67*8q24**	**Positional identification of the rat gene, which led to the identification of mutations in the human gene responsible for MKS3; central nervous system defects are also present in humans and rats**	[273]
Polydactyly (*Lx*)	Zbtb16**,^T^ 8, 52.99 Mb	Skeletal defects and genital hypoplasia	*ZBTB16* 11q23.2	Positional identification of the gene which shows a 2.9 kb deletion in the *Lx* intron 3 and is down-regulated; the heterozygous SHR KO mutant shows anomalies in the caudal part of the body (caudal regression) and growth retardation (the homozygous KO is lethal)	[274, 275]
Pseudoxanthoma elasticum	*Abcc6*^T^ 1, 101.95 Mb	Pseudoxantho-ma elasticum	*ABCC6* 16p13.11	This mineralization disorder is associated with reduced plasma inorganic pyrophosphate; this study of the SD KO mutant points to a critical role of liver ABCC6	[276]
Reed syndrome	*Fh*^T^ 13, 93.65 Mb	Reed syndrome	*FH* 1q43	The SD heterozygous KO mutant shows hematopoietic and kidney dysfunction with kidney anaplastic lesions	[277]
Retinal dystrophy (*Rdy*) (RCS rat)	Mertk*** 3, 121.24 Mb	Retinitis pigmentosa (autosomal recessive)	*MERTK* 2q14.1	Positional identification of the gene: small deletion in the RCS rat, the defect of which could be corrected by gene transfer	[278–280]
Retinal telangiectasia (BN-J rat)	Crb1** 13, 56.27 Mb	Retinal dystrophies (including telangiectasia)	*CRB1* 1q31.3	The BN-J rat shows several retinal abnormalities reminiscent of human macular telangiectasia; sequencing of the BN-J and BN exons revealed the presence of a rearrangement in exon 6 of BN-J, which segregates with the phenotype in an F2 cross	[281]
Retinitis pigmentosa	*Pde6b*^T^ 14, 2.33 Mb	Retinitis pigmentosa (autosomal recessive)	*PDE6B* 4p16.3	The SD KO mutant exhibits photoreceptor degeneration, profound retinal thinning and extensive degeneration of the outer nuclear layer	[282]
Rett syndrome	*Mecp2*^T^ X, 156.65 Mb	Rett syndrome	*MECP2* Xq28	The SD KO mutant shows early motor and breathing abnormalities, growth retardation, malocclusion, reduction of brain weight	[283–285]
Rickets model (type 1A)	*Cyp27b1*^T^ 7, 70.33 Mb	Type 1A rickets	*CYP27B1* 12q14.1	The Wistar KO mutant shows growth failure and rickets; administration of 25-Hydroxyvitamin D3 results in normalization of most phenotypes, including osteogenesis	[286]
Rickets model (type 2A)	*Vdr*^T^ 7q36, 139.34 Mb	Type 2A rickets	*VDR* 12q13.11	The Wistar KO mutant shows growth failure and rickets, with alopecia; a mutant containing the human mutation R270L was also isolated: it also shows rickets symptoms, reversed by 25-Hydroxyvitamin D3 administration; with the above mutant, these 2 mutants could be useful to study the effects of vitamin D derivatives	[286]
Sitosterolemia	Abcg5** 6q12, 7.94 Mb	Sitosterolemia	*ABCG5/* *ABCG8* 2p21	Positional identification of the gene; same missense mutation in SHR, SHRSP and WKY, exhibiting elevated plant sterol accumulation	[287]
Small eye (*rSey*): microphthalmia	Pax6* 3, 95.70 Mb	Aniridia, mental retardation, autism	*PAX6* 11p13	Direct sequencing of the mutant cDNA, which shows a 0.6 kb deletion; impaired migration of neural crest cells; the mutant rat may have some phenotypic component of autism	[288, 289]
Spondylocostal dysostosis (*Oune* mutation)	Tbx6** 1, 198.21 Mb	Spondylocostal dysostosis	*TBX6* 16p11.2	ENU-induced semi-dominant mutation, causing a short and kinked tail and several skeletal abnormalities; positional identification of the mutant gene	[290]
Tenogenesis	*Mkx*^T^ 17, 60.54 Mb	–	–	The Wistar KO mutant shows heterotopic ossification of the Achilles tendon via failed tenogenesis	[291]
Teratoma and infertility (*ter*) in both sexes	Dnd1** 18, 29.61 Mb	–	*–*	Positional identification of the gene: premature stop codon in WKY/Ztm rats; homologous to the mouse mutation *Ter* (which induces testicular teratomas only)	[292]
Testicular feminization (*Tfm*)	Ar* Xq22, 67.66 Mb	Testicular feminization	*AR* Xq12	Direct sequencing of cDNA: single base alteration in the *Ar* gene leads to androgen insensitivity and lack of male sexual development	[222]
T-helper immuno-deficiency (*thid*)	Ptprk** 1, 17.44 Mb	–	*–*	Positional identification of the gene: large deletion in LEC rats, the phenotype of which could be rescued by reconstitution with normal bone marrow cells	[293, 294]
Toothless (*tl*), osteopetrosis	Csf1** 2, 210.52 Mb	–	*–*	Positional identification of the gene: early stop codon in the *tl Csf1* gene*;* similar to the mouse *op*; see below, Polygenic traits, Macrophage development for *Csf1r* KO rats	[295, 296]
Toxicity: aflatoxin B1 toxicity	*Nfe2l2*^T^ 3, 62.50 Mb	–	–	The F344 KO mutant is highly sensitive to aflatoxin B1 toxicity, due to impaired capacity for detoxification; *Nfe2l2* also controls vasculature function: see below	[297]
Toxicity: anthrax toxin susceptibility	Nlrp1** 10q24, 57.69 Mb	–	–	Susceptibility maps in the region of *Nlrp1* (in recombinant inbred strains) and gene polymorphism is correlated with susceptibility in several rat strains; the gene also controls *Toxoplasma* susceptibility: see below	[298]
Toxoplasma susceptibility (*Toxo1*)	Nlrp1*** 10q24, 57.69 Mb	Toxoplasmosis susceptibility	*NLRP1* 17p13.2	Positional identification of the gene; KO of *Nlrp1* in macrophages modifies *Toxoplasma* replication; in humans, association between *NLRP1* polymorphism and toxoplasmosis susceptibility; the gene also controls sensitivity to anthrax toxin: see above	[299]
Tremor (tremor rat: TRM/Kyo, carrying the *tm* mutation)	Aspa*^,T^ 10, 59.84 Mb	Canavan disease	*ASPA* 17p13.2	Positional identification of a deletion spanning > 200 kb in the TRM/Kyo rat; injection of N-acetyl-L-aspartate, the *Aspa* precursor, induces absence-like seizure in normal rats (the tremor rat exhibits absence-like seizure); an F344 KO mutant shows abnormal myelination but no tremor; however an *Aspa*/*Hcn1* double mutant shows tremor, like the TRM/Kyo rat, which is a double mutant (see below, Polygenic traits, Epilepsy, tremor, *Hcn1*); the pathogenesis of tremor involves ionotropic glutamate receptors	[124, 300, 301]
Tremor: Zitter rat (*zi* mutation)	Atrn*** 3q35, 123.43 Mb	–	–	*zi* induces hypomyelination and vacuolation in the CNS; positional identification of the gene; *zi* is homologous to the mouse *mg* (mahogany); complementation by transgenic membrane-type *Atrn*	[302, 303]
Tremor: VF rat (*vf* mutation)	Dopey1** 8, 94.12 Mb	–	–	*vf* induces hypomyelination and vacuolation in the CNS; positional identification of the gene, which carries a nonsense mutation	[304]
Tremor (*Trdk* mutation)	Kcnn2** 18, 39.33 Mb	–	–	ENU-induced missense mutation; positional identification of the mutant gene	[305]
Unilateral renal agenesis (URA; *Renag1*)	Kit*** 14, 37.07 Mb	–	–	ACI rats exhibit URA and white spotting; positional identification of the gene, which carries an insertion; removing the insertion by the CRISPR/Cas9 system corrects the disease and the white spotting phenotype; *Kit* also controls the hooded phenotype: see above, Coat color; the gene may also control anemia (see above)	[306, 307]
Warfarin resistance (*rw*)	Vkorc1** 1, 199.34 Mb	VKCFD2 and warfarin resistance	*VKORC1* 16p11.2	Positional identification of the gene, mutated in warfarin resistance (humans and rats) and VKCFD2 (humans)	[308, 309]
Wilson disease model	Atp7b** 16q12, 74.87 Mb	Wilson disease	*ATP7B* 13q14.3	Positional identification of the gene: deletion in the LEC rat gene, causing hepatitis	[310, 311]
Wolfram disease model	*Wfs1*^T^ 14, 78.64 Mb	Wolfram disease	*WFS1* 4p16.1	The SD KO mutant shows the core symptoms of the human disease: diabetes mellitus, glycosuria, neurodegeneration; treatment with a GLP1 receptor agonist prevents the development of diabetic phenotype in the KO rat	[312, 313]
Wolman disease model (Wolman rat)	Lipa* 1, 252.82 Mb	Wolman disease	*LIPA* 10q23	Direct sequencing of the mutant rat cDNA: deletion of the *Lipa* gene in the Wolman rat	[314]
**B) Polygenic traits** (*QTL symbol*)					
Addiction: alcohol consumption	Adcyap1r1* 4, 85.66 Mb	Alcohol consumption in women	ADCYAP1R1 7p14.3 (Association study)	Positional identification of the gene and expression studies in congenic strains; the trait is female-specific; *Adcyap1r1* is upregulated in alcohol-preferring females and its promoter contains several ERE’s and polymorphisms associated with a differential response to estrogen stimulation in vitro	[315]
Addiction: alcohol consumption	Grm2* 8, 115.34 Mb	–	–	Positional identification of the gene; stop codon in the alcohol-preferring rat strain allele; (see also above, Monogenic traits, Addiction; opioid consumption); however, this conclusion was challenged on the basis of experiments showing that a lentiviral-delivered short-hairpin RNA-mediated KO of *Grm2* does not promote alcohol drinking	[316–318]
Addiction: alcohol consumption (*Alc22*)	Crhr2* 4, 85.29 Mb	–	–	Polymorphisms in the promoter, coding region, and 3’UTR were associated with altered CRHR2 binding density in alcohol-preferring rat strain (no mapping of the trait)	[319]
Addiction: alcohol consumption (*Alc11/13*)	Cyp4f18** 16, 19.50 Mb	–	–	DNA sequencing of rats from HS-derived high- and low-alcohol-drinking lines revealed several genomic regions showing signature of selection, including genes located in previously identified QTLs^d^	[320]
Addiction: alcohol consumption (*Alc11/13*)	Fam129c** 16, 20.03 Mb	–	–	See comment above, on *Cyp4f18*	[320]
Addiction: alcohol consumption (Alc*5/9/12*)	Grin2a** 10q11, 5.71 Mb	–	–	See comment above, on *Cyp4f18*	[320]
Addiction: alcohol consumption (*Alc11/13*)	Myo9b** 16, 19.67 Mb	–	–	See comment above, on *Cyp4f18*	[320]
Addiction: alcohol consumption	*Npy*^T^ 4, 79.56 Mb	–	–	*Npy* deletion in an alcohol non-preferring rat model elicits differential effects on alcohol consumption and body weight	[321]
Addiction: alcohol consumption (*Alc11/13*)	Pgls** 16, 20.02 Mb	–	*–*	See comment above, on *Cyp4f18*	[320]
Adiposity	*Angptl8*^T^ 8, 22.86 Mb	–	*–*	The F344 KO mutant shows lower body weight, lower fat content and lower triglyceride levels, but higher heart lipase levels than WT rats	[322]
Allergic rhinitis	*Muc1*^T^ 2, 188.54 Mb	–	*–*	The SD KO rat shows aggravation of allergic rhinitis and suppression of expression of epithelial cell connection proteins	[323]
Angiogenesis	*Agtr1a*^T^ 17q12, 35.90 Mb	–	*–*	The SS KO rat shows no response to injection of angiotensin II (as expected) and no increase in hind limb vessel density upon angiotensin-(1–7) infusion (the effects of which are mediated by *Mas1*), indicating an AGTR1A-MAS1 interaction; the gene also controls Blood pressure (see below)	[324]
Angiogenesis	Wars2**^, T^ 2q34, 201.17 Mb	Cardio-metabolic phenotypes	*WARS2* 1p12	Positional identification of the gene controlling coronary flow; the BN KO mutant shows diminished cardiac capillary density and reduced coronary flow; the gene also controls the metabolic syndrome: see below	[325]
Aorta elastic tissue integrity (*Vetf3*)	Pi15** 5, 0.79 Mb	–	–	High resolution mapping in a HS; lower expression of *Pi15* in the susceptible strain BN (combined with higher expression of a long intergenic noncoding RNA)	[326]
**Arthritis**	CIIta** **10, 5.21 Mb**	**RA, MS, myocardial infarction**	***CIITA*** **16p13**	**Positional identification of the rat gene, definitively identified by sequencing and expression analysis; in humans, polymorphism in the promoter was associated with disease susceptibility**	[327]
**Arthritis** **(*Pia7, Oia2*)**	Clec4b** **4q42,** **156.11 Mb**	**RA**	***CLEC4A*** **12p13**	**Positional identification of the*Aplec*rat gene complex and then of the*Clec4b*gene; association was found between RA and*CLEC4A*(=*DCIR*) in human patients**	[328–331]
Arthriris	*Fcgr1a*^T^ 2, 198.43 Mb	–	*–*	A conditional SD KO rat was generated in which the gene was inactivated in the dorsal root ganglion; the rat shows attenuation of pain upon antigen-induced arthritis, indicating that neuronal FCGR1A contributes to arthritic pain	[332]
Arthritis	*Git2*^T^ 12, 47.59 Mb	–	*–*	The SD KO rat with induced arthritis shows a more severe disease, with decreased collagen II expression and increased expression of inflammatory cytokines	[333]
Arthritis: gout	*P2ry14*^T^ 2, 149.33 Mb	–	*–*	The SD KO rat shows disruption of urate-induced histopathologic changes in rat synoviums, accompanied with a significant inhibition of pyroptotic macrophage death	[334]
Arthritis: PIA	Hip1** 12q16, 24.18 Mb	–	*–*	Positional identification of the gene, which is required for the increased invasiveness of synoviocytes from arthritic rats and from RA patients	[335]
Arthritis (P*ia8*)	Il22ra2**			See *Eae29*	
**Arthritis (*Pia4*)**	Ncf1** **12, 25.50 Mb**	**RA**	***NCF4*** **22q13.1**	**Positional identification of the gene and of the causative mutation; the gene controls the production of reactive oxygen species; see also below: Experimental autoimmune neuritis**	[43, 330, 336, 337]
Arthritis: PIA	Lta, Ltb, Tnf, Lst1, Ncr3** 20p12, 3.65–3.71 Mb	–	*–*	Positional identification of a recombination-resistant 33 kb segment, made of 5 genes, within the MHCIII region; one conserved haplotype regulates arthritis; haplotype-specific differences in gene expression and alternative splicing correlate with susceptibility to arthritis; the haplotype specifically regulates adjuvant-induced arthritis, but not antigen-induced autoimmunity	[338, 339]
Arthritis: *Pia1*	RT1-Ba** 20p12, 4.07 Mb and RT1-Bb** 20p12, 4.04 Mb	RA	*MHCII* 6p21.32	Using a mixed genetic and functional approach, these 2 genes (orthologs of the human *HLA-DQA* and *HLA-DQB* loci, in the MHCII region) were shown to control the onset and severity of PIA	[340]
**Arthritis**: PIA	Vav1** **9q12, 9.62 Mb**	**RA**	***VAV1*** **19p13.2**	**Polymorphism in*Vav1*controls PIA in the rat; in humans,*VAV1*SNPs are associated with RA; see also below,*Eae4***	[341]
Asthma	*Trpa1*^T^ 5, 3.78 Mb	–	*–*	The SD KO rat is largely protected from immune cell infltration into bronchoalveolar lung fuid in the ovalbumin model of asthma; on the other hand, it shows normal behavioral responses in multiple models of pain and itch	[342]
Behavior	*Cplx1*^T^ 14, 2.20 Mb	–	*–*	The SD KO mutant shows severe ataxias and tremor, dystonia, uncoordinated locomotion, exploratory deficits, anxious behavior and sensory deficits as well as decreased dendritic branching in spinal motor neurons	[343]
Behavior	*Phf24*^T^ 5, 58.36 Mb	–	*–*	The F344 KO mutant shows no apparent changes in gross behaviors during adolescence but, at older age, it exhibits elevated spontaneous locomotor activity, emotional hyper-reactivity, reduced anxiety behaviors and cognitive deficits; it also shows a higher sensitivity to induced convulsive seizures	[344]
Behavior: Attention-deficit hyperactivity disorde rmodel	*Adgrl3*^T^ 14, 28.36 Mb	Attention-deficit hyperactivity disorder	*ADGRL3* 4q13.1	The SD KO mutant shows persistent hyperactivity, increased acoustic startle, reduced activity in response to amphetamine and female-specific reduced anxiety-like behavior; dopamine signaling is dysregulated in the neostriatum	[345, 346]
Behavior: aggressive phenotype	*Tph2*^T^ 7, 58.04 Mb	–	*–*	The DA KO mutant exhibits (as expected) profoundly diminished serotonin level and displays increased aggressiveness	[347]
Behavior: anxiety	Cckar* 14, 59.61 Mb	–	*–*	Gene deletion in the OLETF rat; no mapping of the trait; see also below, Body temperature and Diabetes, type 2	[348]
**Behavior: anxiety, depression**	Ctnnd2** **2, 83.39 Mb**	**Schizophrenia, Depressive disorder**	***CTNND2*** **5p15.2**	**Positional identification of the rat gene; the human gene was then associated with schizophrenia and major depressive disorder**	[28, 349, 350]
Behavior: anxiety, depression	*Slc6a4*^ENU^ 10, 63.15 Mb	Anxiety/ depression	*SLC6A4* 17q11.2	The Wistar KO mutant lacking the serotonin transporter shows anxiety, depression-related behavior and impaired object memory as well as alterations in DNA methylation of the urocortin promoter	[351, 352]
Behavior: anxiety, drug addiction	*Oprl1*^ENU^ 3, 177.23 Mb	–	*–*	The Wistar KO mutant lacking the nociceptin/orphanin FQ receptor rat shows an anxiety-like phenotype and is more sensitive to the rewarding effect of morphin	[353, 354]
Behavior: autism-like symptoms	*Nrxn1*^T^ 6, 14.75 Mb	Autism	*NRXN1* 2p16	The SD KO mutant shows persistent nonsocial deficits, including hyperactivity, deficits in simple instrumental learning, latent inhibition, and spatial-dependent learning	[355]
Behavior: dopamine-related brain disorders	*Drd1*^ENU^ 17, 11.10 Mb	–	*–*	The Wistar mutant carries a missense mutation that leads to a decreased transmembrane insertion of DRD1; it displays normal basic neurological parameters and locomotor activity but reduced social cognition (such as social interaction)	[356]
Behavior: dopamine-related brain disorders	*Slc6a3*^ENU*,*T^ 1, 32.32 Mb	Several psychiatric disorders	*–*	Two mutants are available: an F344 ENU-induced missense mutant and a targeted Wistar KO mutant; both strains show locomotor hyperactivity and impaired cognitive processes; they are excellent models for the evaluation of the effects of novel therapeutics on cognitive functions linked to the dopamine transporter	[357, 358]
Behavior: drug addiction (cocaine)	*Trpc4*^T^ 2, 143.43 Mb	–	*–*	The F344 KO mutant shows reduced acquisition of cocaine self-administration compared to WT rats; see also below: Blood pressure -PAH- and Behavior, drug addiction	[359]
Behavior: fear memory	*Crebbp*^T^ 10, 11.59 Mb	–	*–*	An SD mutant was generated using a novel method that targets the *Crebbp* gene in a population of neurons of the medial prefrontal cortex; the mutant shows impaired fear (remote) memory and impaired extinction learning	[360]
Behavior: fear memory	*Rin1*^T^ 1, 220.33 Mb	–	*–*	The SD KO mutant shows a deficit in the formation and extinction of auditory fear memory, associated with enhanced apoptosis in the hippocampus	[361]
Behavior: fear memory and coping	*Nr3c1*^T^ 18p12, 31.73 Mb	–	*–*	A conditional SD KO mutant was generated, targeting output neurons and the prelimbic cortex; females exhibit deficits in acquisition and extinction of fear memory; males exhibit enhanced active-coping behavior during forced swim	[362]
Behavior: mental illnesses	*Disc1*^T^ 19, 57.82 Mb	Mental illnsesses	*DISC1* 1q41.2	The SD mutant shows changes in white matter microstructural integrity and deficits in neurite density (it recapitulates many of the neuroimaging findings seen in populations of schizophrenia); the male is more affected than the female mutant	[363]
Behavior: neuropsychiatric disorders model	*Cacna1c*^T^ 4, 150.64 Mb	Autism, bipolar disorder, schizophrenia	*CACNA1C* 12p13.33	The heterozygous SD KO mutant shows deficits in social behavior and in pro-social ultrasonic communication; however this haploinsufficiency has a minor positive impact on memory functions	[364, 365]
Behavior: neuropsychiatric disorders and PCH3 model	*Pclo*^T^ 4, 16.45 Mb	Psychiatric and developmental disorders and PCH3	*PCLO* 7q21.11 (GWAS)	The SD KO mutant shows a reduction in the number of synaptic vesicles, which show abnormal recycling, deficient formation of early endosomes and altered synaptic transmission; the mutant displays impaired motor coordination	[366, 367]
Behavior: stress response	*Dpp4*^T^ 3, 48.29 Mb	–	*–*	The DA.F344 KO congenic mutant is stress-resilient and shows decreased expression of *Nr3c1* and *Fkbp5* in the amygdala and the hypothalamus as well as lower stress-induced peripheral corticosterone levels	[368]
Behavior: stress response	*Nr4a1*^T^ 7, 142.90 Mb	–	*–*	The male FHH KO mutant was only used to study gene expression in the prefrontal cortex; the mutant shows reduced expression of the AMP-activated protein kinase, indicating that NR4A1 favors the adverse effects of stress; the gene also controls Parkinson disease (see above) and Renal injury (see below)	[369]
Behavior: stress response	*Nrg1*^T^ 16, 62.97 Mb	Schizophrenia	*NRG1* 8p12	The F344 KO mutant shows alterations in HPA axis activity and behavioral responses to stress	[370]
Behavior: stress response (*Stresp24*)	Stim1** 1, 167.37 Mb	Autoimmunity	*STIM1* *11p15.4*	Positional identification of the gene; nonsense mutation in several SHRSP substrain alleles, absent in WKY and other normotensive strains; this mutation impairs Ca^++^ signaling in astrocytes; this pleiotropic genes also controls Diabetes insipidus (see above), Renal injury and Stroke (see below)	[371, 372]
Bladder function	*Trpv4*^T^ 12, 47.70 Mb	–	*–*	The phenotype of the SD KO mutant shows that in a model of underactive bladder, intravesical activation of TRPV4 improves bladder function	[373]
Blood pressure	*Agtr1a*^T^ 17q12, 35.91 Mb	–	*–*	The MSH6 KO mutant shows an extremely high blood pressure-like phenotype; the gene also controls Angiogenesis (see above)	[239]
**Blood pressure:*BpQTL2***	Adamts16**^,T^ **1, 36.47 Mb**	**Hypertension**	***ADAMTS16*5p15**	**Positional identification of the gene, which shows exonic variants; association between ADAMTS16 and blood pressure was then discovered in humans; KO of the gene in the SS rat leads to lower blood pressure; this gene also controls male fertility: see above, Monogenic traits, Infertility**	[374, 375]
**Blood pressure**	Add1** **14, 82.06 Mb**	**Hypertension and CV risks**	***ADD1*** **4p16.3**	**Positional identification of the gene: missense polymorphisms in the Milan Hypertensive Rat and in humans; in vitro functional studies**	[376, 377]
**Blood pressure:*Bp77***	Arntl** **1, 171.06 Mb**	**Hypertension and NIDDM**	***ARNTL*** **11p15**	**Functional polymorphisms found in the rat gene promoter; association was then established in the human with blood pressure and type 2 diabetes**	[378]
Blood pressure	*Cd247*^T^ 13q23, 88.88 Mb	Hypertension	1q24 locus (*GPA33, CD247, F5, REN)*	The SS KO mutant exhibits reduced kidney infiltration of T cells, mean arterial blood pressure and kidney damage	[379, 380]
Blood pressure	Cd36** 4, 14.15 Mb	–	–	Positional identification of the gene, combined with gene expression studies; deficient renal expression of *Cd36* (in SHR) is a genetically determined risk factor for spontaneous hypertension; the gene also controls diabetes: see below	[29]
Blood pressure (*C17QTL1*)	Chrm3**^,T^ 17q12, 63.99 Mb	–	*–*	Positional identification of the gene; the SS rat carries a missense mutation enhancing receptor activity; the KO SS mutant exhibits lower salt-induced hypertension and improved renal function	[381]
Blood pressure	Chst12** 12, 18.19 Mb	Hypertension	*7p22*	Positional identification of the gene; the SS allele contains mutations when compared with several normotensive strains; this rat region is homologous to a region on human chromosome 7 that has been linked to blood pressure	[382]
Blood pressure	*Clcn6*^T^ 5, 168.47 Mb	Hypertension	*AGTRAP-PLOD1* locus; 1p36	The SS KO mutant shows decreased blood pressure; the human locus was identified in GWAS and *CLCN6* could be linked to blood pressure and renal phenotypes	[39]
Blood pressure	Cyp11b1** 7, 112.98 Mb	–	*–*	Positional identification of the gene; the characteristic steroid profiles of SS and SR rats can be explained by the biochemical properties of CYP11B1; 5 mutations found in the SS allele, segregating with blood pressure and altered steroid biosynthesis in a SS X SR cross	[383]
Blood pressure	Cyp17a1** 1q55, 266.42 Mb	Hypertension	*CYP17A1* 10q24.32	Extensive proteomics and transcriptome studies in the BN and SHR strains led to the discovery that *Cyp17a1* is downregulated in SHR, probably as a consequence of a promoter mutation; in humans a SNP in *CYP17A1* was associated with hypertension	[384]
Blood pressure	Gja8** 2, 199.05 Mb	–	*–*	The *Gja8* mutation present in the SHR-Dca strain (causing cataract; see above, Monogenic traits) lowers blood pressure and decreases high density lipoprotein cholesterol concentration	[385]
Blood pressure	*Gper1*^T^ 12, 17.31 Mb	–	*–*	The SS KO mutant presents with lower blood pressure, accompanied by altered microbiota and improved vascular relaxation	[386]
Blood pressure	*Hsd11b2*^T^ 19q12, 37.48 Mb	SAME	*HSD11B2* 16q22.1	The F344 KO mutant exhibits hypertension, hypokalemia, renal injury; the phenotype closely models the human SAME	[387]
Blood pressure	*Htr7*^T^ 1, 254. 55 Mb	–	*–*	Unlike wild-type rats, the SD KO mutant does not show reduced mean arterial pressure nor splanchnic venodilation upon serotonin infusion	[388]
Blood pressure	*Kcnj1*^T^ 8, 33.45 Mb	Type II Bartter syndrome	*KCNJ1* 11q24	The SS KO mutant exhibits protection from salt-induced blood pressure elevation	[389]
Blood pressure	*Kcnj16*^T^ 10, 99.33 Mb	Brugada syndrome (arrhythmias)	*KCNJ16* 17q24.3	The SS KO mutant exhibits hypokalemia and reduced blood pressure; when fed on a high salt diet, this mutant dies as a result of salt wasting and severe hypokalemia; the gene also controls pH homeostasis: see above, Monogenic traits, Acidosis	[390]
Blood pressure	Ncf2***,^T^ 13, 75.2 Mb	–	–	Positional identification of the gene, which shows higher expression and promoter mutation in the SS rat; disruption of the gene reduces hypertension and renal oxidative stress and injury; *Ncf2* is involved in luminal flow-mediated O_2_^·−^ production (i.e. oxidative stress)	[391, 392]
Blood pressure	*Nox4*^T^ 1, 150.80 Mb	–	*–*	The SS KO mutant shows reduction of salt-induced hypertension and of albuminuria compared with the wild-type SS rat; *Nox4* contributes to the production of H_2_0_2_ (i.e. oxidative stress)	[392, 393]
Blood pressure	*Nppa*^T^ 5q36, 165.81 Mb	Hypertension	*AGTRAP-PLOD1* locus; 1p36	The SS KO mutant shows increased blood pressure; the human locus had been identified in GWAS and *NPPA* could be linked to blood pressure phenotypes	[39]
Blood pressure	*Nppb*^T^ 5q36, 164.79 Mb	Hypertension and left ventricular dysfunction	*NPPB* 1p36.22	The SS KO mutant shows adult-onset hypertension, left ventricular hypertrophy and increased cardiac stiffness	[394]
Blood pressure	*Nr2f2*^T^ 1, 131.45 Mb	Hypertension	*NR2F2* 15q26	*NR2F2* was associated with hypertension in humans; an hypomorphic SS mutant shows lower systolic and diastolic blood pressures	[395]
Blood pressure	Pappa2** 13, 36.39 Mb	–	–	Positional identification of the gene (including generation of SS subcongenic strains); renal cortex *Pappa2* mRNA level is lower in the SS rat	[396]
Blood pressure (HTNB)	*Pde3a*^T^ 4, 175.43 Mb	Hypertension (HTNB)	*PDE3A* 12p12.2	An SD mutant carrying a 9 bp deletion similar to that found in a human patient is hypertensive and recapitulates HTNB (including shorter fingers); the mutation causes an increase in enzyme activity with peripheral vascular resistance; the data suggest that soluble guanylyl cyclase activation could be suitable for the treatment of HTNB patients	[46]
Blood pressure	*Plekha7*^T^ 1, 185.43 Mb	Hypertension	*PLEKHA7* 11p15.1	*PLEKHA7* is a candidate gene for human hypertension; the SS KO mutant shows attenuated salt-sensitive hypertension and vascular improvements	[397]
Blood pressure	*Plod1*^T^ 5, 168.38 Mb	Hypertension	*AGTRAP-PLOD1* locus 1p36	The SS KO mutant shows increased systolic blood pressure; the human locus was identified in GWAS	[39]
Blood pressure	*Prdx2*^T^ 19, 26.08 Mb	–	*–*	The SHR KO mutant exhibits shorter life span and modest blood pressure increase via increased oxidative stress	[398]
Blood pressure	*Rag1*^T^ 3, 97.87 Mb	SCID	*RAG1* 11p13	The SS KO mutant exhibits attenuation of blood pressure and of renal damage (and lymphocyte depletion: see above, Monogenic traits, Immunodeficiency)	[399]
Blood pressure	*Rarres2*^T^ 4, 78.21 Mb	–	–	The SD KO female mutant (but not the KO male) exhibits a relative resistance to hypertension in response to a hypertensive challenge	[400]
Blood pressure	*Ren*^T^ 13q13, 55.55 Mb	–	–	The SS KO mutant shows a greatly reduced blood pressure, changes in kidney morphology and reduced adrenal synthesis of aldosterone and Cyp11b2	[401, 402]
Blood pressure	*Resp18*^T^ 9, 82.47 Mb	–	–	The SS KO mutant shows increased systolic and diastolic blood pressure, as well as increased renal damage (*Resp18* is located in a blood pressure QTL)	[403]
Blood pressure	*Sh2b3*^T^ 12, 40.26 Mb	Hypertension	*SH2B3* 12q24	*SH2B3* has been associated with hypertension; in the SS KO mutant, hypertension and renal disease are attenuated via inflammatory modulation; the gene also controls cardiac inflammation: see above, Monogenic traits	[404]
Blood pressure	Sry1* Y	Hypertension	? Y	Delivery of *Sry1* cDNA to the kidney increases blood pressure in normotensive WKY rats	[405]
Blood pressure	Zbtb16**^T^ 8, 51.57 Mb	–	–	Positional identification of the gene in RI strains and in an SHR-PD congenic; deletion in the intron 2 of the PD allele, which is down-regulated and is protective; the heterozygous SHR KO mutant shows no change in blood pressure (the homozygous KO is lethal)	[406, 407]
Blood pressure: captopril effects	Ednrb** 15q22, 88.00 Mb	–	*–*	The antihypertensive effects of the ACE inhibitor captopril behave as a polygenic trait in RI strains; *Ednrb* was positionally identified: correlation between renal expression and captopril effects; this gene also controls aganglionosis (see above, Monogenic traits)	[408]
Blood pressure: PAH	*Ddah1*^T^ 2, 251.63 Mb	–	*–*	The SD KO mutant shows no specific phenotype under control conditions, but exhibits exacerbated monocrotaline-induced PAH, lung fibrosis as well as right ventricule hypertrophy and dysfunction	[409]
Blood pressure: PAH	*Kcnk3*^T^ 6, 27.15 Mb	PAH	*KCNK3* 2p23.3	The SD KO mutant shows predisposition to vasoconstriction of pulmonary arteries, strong alteration of right ventricular cardiomyocyte excitability and develops age-dependent PAH	[410]
Blood pressure: PAH	*Park7*^T^ 5, 167.98 Mb	Familial PD (recessive)	*PARK7* 1p36.23	The KO mutant shows a worse degree of PAH than WT rats under hypoxia; the gene also controls Parkinson disease: see above, Monogenic traits	[411]
Blood pressure: PAH	Slc39a12**^,T^ 17, 81.46 Mb	–	–	Positional identification of the gene [WKY rats exposed to hypoxia show increased expression of *Slc39a12* (ZIP12 protein)]; the KO WKY mutant shows attenuation of PAH	[412]
Blood pressure: PAH	*Sod3*^T^ 14, 61.07 Mb	–	–	In the SS KO mutant, the mutation favors PAH and subsequent RV hypertrophy under stress conditions	[413]
Blood pressure: PAH	*Trpc4*^T^ 2, 143.43 Mb	–	–	The F344 KO mutant shows reduced severity of pulmonary arterial occlusions and survival benefit in severe PAH (the gene is also involved in Pain, see below and Behavior, drug addiction: see above)	[414]
Blood pressure and QT-interval	Rffl-lnc1*** 10, 71.07 Mb	QT-interval	17q12 (*RFFL* region)	Positional identification of the gene; the LEW allele contains a 19 bp deletion in the long non-coding RNA (5’UTR of *Rffl*), which increases blood pressure and shortens QT-interval relative to the SS rat (“cryptic allele”); the normal phenotypes were rescued by a specific targeted 19 bp insertion in the LEW allele	[33]
Body temperature	Cckar* 14, 59.61 Mb	–	*–*	Gene deletion in the OLETF rat (no mapping of the trait): the gene seems also involved in diabetes and behavior; see above, Behavior, anxiety and below Diabetes type 2	[415, 416]
Body weight (muscle mass)	*Mstn*^T^ 9, 53.31 Mb	–	*–*	SS and SD KO mutants were studied; they show marked increases in muscle mass and lower fat content	[417, 418]
Body weight (liver mass)	*Ogdh*^T^ 14, 86.41 Mb	Hypotonia, metabolic acidosis	*OGDH* 7p13	The SD KO heterozygous mutant shows increased liver weight; high fat diet results in liver dysfunction (homozygous mutants are lethal)	[419]
Bone growth	*Cftr*^T^ 4q21, 42.69 Mb	Cystic fibrosis	*CFTR* 7q31.2	Young SD KO rats do not develop lung or pancreatic disease; however, they show a defect in linear bone growth and bone health that is attributed to IGF-1 deficiency (for Cystic fibrosis, see above, Monogenic traits)	[420]
Bone growth	*Nppc*^T^ 9, 93.73 Mb	Short stature	*NPPC* 2q37.1	The F344 KO mutant exhibits a deficit in endochondral bone growth and growth retardation	[421]
Bone structure and function	*Bglap*^T^ 2, 87.74 Mb	–	*–*	The SD KO mutant shows increased trabecular thickness, density and volume, and increased bone strength	[422]
Brain development	*Bscl2*^ENU^ 1, 225.04 Mb	Congenital generalized lipodystrophy	*BSCL2* 11q12.3	The KO mutant shows a slightly decreased brain weight and impairment of spatial working memory; see also above, Monogenic traits, Lipodystrophy, and Infertility	[223]
Brain injury	*Aqp4*^T^ 18, 6.77 Mb	–	*–*	Following subarachnoid hemorrhage, the KO mutant shows increased water content in the whole brain, which aggravates the neurological deficits through impairment of the glymphatic system.	[423]
Brain injury: acute cerebral infarction	*Uba6*^T^ 14, 23.51 Mb	–	*–*	The SD conditional KO mutant specifically lacks expression of the gene in the brain and shows aggravation of cerebral infarction, accompanied by increased level of apoptosis	[424]
Cancer, colon	Rffl or Rffl-lnc1* 10, 70,16 Mb or 71.07 MB	–	*–*	Positional identification of the gene(s); higher expression of *Rffl* in S-LEW congenic rats, which also show higher expression of *Mbd2* and higher susceptibility to colorectal carcinogenesis; see above, Blood pressure and QT-interval	[425]
Cancer, esophageal carcinoma	*Mir31*^T^ 5, 107.21 Mb	–	*–*	The SD KO mutant is resistant to zinc-deficiency associated esophageal carcinoma as a result of a strong reduction in an inflammatory process	[426]
Cancer, mammary (*Mcs1a*)	Putative regulatory site** 2, ~ 6.50 Mb	–	*–*	Positional identification of the locus; cancer resistance is associated with increased expression of the nearby gene *Nr2f1;* the human homologous region (5q11-q34) is frequently deleted in breast cancers	[427]
Cancer, mammary (*Mcs1b*)	Mier3** 2, 62.31 Mb	Breast cancer risk locus	*MAP3K1* or *MIER*3 5q11.2	Positional identification of the gene; higher expression in mammary glands of susceptible females	[428]
**Cancer, mammary (*Mcs5a1*)**	Fbxo10** **5, 60.59 Mb**	**Breast cancer risk locus**	***FBXO10 (MCS5A1)*** **9p13**	**Positional identification of the gene; up-regulation in T cells is associated with susceptibility; causal SNVs are probably stress-responding regulatory sites**	[429, 430]
**Cancer, mammary (*Mcs5a2*)**	Frmpd1** **5, 60.75 Mb**	**Breast cancer risk locus**	***FRMPD1 (MCS5A2) 9p13***	**Positional identification of the gene; up-regulation in the spleen was associated with cancer resistance**	[430]
Cancer, mammary (*Mcs5c*)	Regulatory site** 5, ~ 81 Mb	–	–	Positional identification of the locus*; Msc5c* is located in a gene desert and regulates expression of the neighboring gene *Pappa1* during a critical mammary developmental time period	[431, 432]
Cancer, mammary (*Mcs30*)	Fry* 12, 7.68 Mb	–	–	Positional identification of the gene; several SNPs between F344 (susceptible) and COP (resistant); decreased expression of FRY in human cancers	[433]
Cancer, mammary gland development	*Cdkn1b*^T^*,* 4, 168.69 Mb	Multiple endocrine neoplasia type 4	*CDKN1B* 12p13.1	In humans the frequency of a population of quiescent *CDKN1B* expressing cells was associated with breast cancer risk; the *Cdkn1b* KO ACI rat shows increased proliferation and pregnancy-associated changes in the mammary gland; *Cdkn1b* could impact mammary cancer risk; see also above, Monogenic traits, Cancer, multiple endocrine neoplasia and Obesity	[83]
Cardiac mass	*Cfb*^*T*^	–	–	See below, Metabolic syndrome	[434]
Cardiac mass (*Cm10*)	Endog** 3, 8.74 Mb	–	–	Positional identification of the gene, which is underexpressed in strains with increased cardiac mass; exonic mutation in SHR; *Endog* seems to be implicated in mitochondrial physiology	[435]
**Cardiac mass (LVM)**	Ogn** **17, 14.61 Mb**	**LVM**	***OGN*** **9q22.31**	**Localization of a QTL and genome-wide gene expression studies associated upregulation of*Ogn*(due to sequence variation in the*Ogn*3′ UTR) with elevated LVM; this finding was translated to humans**	[436]
Cardiac mass, fibrosis	Zbtb16**^,T^ 8, 51.57 Mb	–	–	Positional identification of the gene in RI strains and in an SHR-PD congenic: deletion in the intron 2 of the PD allele, which is down-regulated and is protective; the heterozygous SHR KO mutant shows reduced cardiomyocyte hypertrophy and interstitial fibrosis (the homozygous KO is lethal)	[406, 407]
Cholesterol level and hepatic steatosis (*Hpcl1*)	Srebf1*** 10, 46.33 Mb	Cholesterol level and IFAP	*SREBF1* 17p11.2	Positional identification of the gene; the SHR allele is associated with deficient expression of mRNA and protein; an SHR transgenic strain shows restoration of hepatic cholesterol level	[437]
Chronic kidney disease(CKD)	*Mir146b (5p)*^T^ 1, 266.09 Mb	–	–	CKD contributes to secondary cardiovascular impairment (cardiorenal syndrome type 4); in the surgical excision model of 5/6 nephrectomy, the KO SD female mutant shows sex-specific exacerbated renal hypertrophy and fibrosis with renal dysfunction yet lower blood pressure and less pronounced cardiac remodeling	[438]
Chronic kidney disease(CKD)	*Sod3*^ENU^ 14, 60.96 Mb	–	–	The SS mutant develops profound CKD characterized by focal necrosis and fibrosis, glomerulosclerosis, massive proteinaceous cast accumulation with tubular dilatation, interstitial fibrosis with hypertension and renal failure; see also below, Vascular function	[439]
**Diabetes, type 1: T1DM (*Kdp1*)**	Cblb*** **11, 51.04 Mb**	**Diabetes, type 1**	***CBLB*** **3q13.11**	**Positional identification of the gene, mutated in the Komeda diabetes-prone rat; complementation with the WT gene significantly suppressed the phenotype of the KDP rats**	[440]
Diabetes, type 1: T1DM (*Iddm8*)	Dock8** 1, 242.93 Mb	–	*–*	Positional identification of the gene which harbors a missense mutation in the diabetic LEW.1AR1/Ztm-*idmm* rat	[441]
Diabetes, type 1: T1DM Lymphopenia (*Iddm2/lyp*)	Gimap5** 4, 78.38 Mb	Systemic lupus erythematosus	*GIMAP5* 7q36.1	Positional identification of the gene, mutated in the diabetes-prone BB rat; lymphopenia is essential for the development of the diabetic phenotype; in humans, *GIMAP5* could play a role in the pathogenesis of systemic lupus erythematosus	[442–444]
Diabetes, type 1: T1DM (*Iddm37*)	Ubd*, ^T^ 20, 1.87 Mb	–	–	Positional identification of *Ubd*, the promoter of which is polymorphic; high UBD expression is associated with virus-induced T1DM susceptibility (LEW.1WR1 rat for instance); an LEW.1WR1 KO rat shows reduced susceptibility, confirming the role of *Ubd*; the gene also controls cardiac ischemia (see above)	[445]
Diabetes, type 1: T1DM	*Ifnar1*^T^ 11, 31.64 Mb	T1DM	Several genes acting downstream *IFNAR1*	Two LEW.1WR1 KO mutants were isolated; they exhibit, as expected, an impaired response to interferon I treatment; they are partially protected against virus-induced diabetes	[446]
**Diabetes, type 2: T2DM**	Adra2a** **1, 274.77 Mb**	**Increased T2DM risk**	***ADRA2A*** **10q25.2**	**Positional identification of the gene, overexpressed in the diabetic Goto-Kakizaki rat, mediating adrenergic suppression of insulin secretion; association was then found between*ADRA2A*and increased T2DM risk in humans**	[447]
Diabetes, type 2: T2DM	*Abcc8*^T^ 1, 102.11 Mb	T2DM and Hyperinsulinemic hypoglycemia and	*ABCC8* 11p15.1	The SD KO mutant is glucose intolerant and shows enhanced insulin sensitivity; T2DM was induced in this mutant which was then treated with glimepiride (a sulfonylurea); the treatment decreased blood glucose levels, suggesting an extra-pancreatic, direct effect on insulin-sensitive tissues	[448, 449]
Diabetes, type 2: T2DM (*Odb2*)	Cckar** 14, 59.61 Mb	–	–	Positional identification of the gene, deleted in the OLETF rat; mapping studies suggest an interaction with an X-linked QTL; the gene might also control pancreatic duct hyperplasia; see also above, Body temperature and Behavior, anxiety	[450, 451]
**Diabetes: T2DM (Insulin resistance and hyperlipidemia)**	Cd36*** **4, 14.15 Mb**	**T2DM: Insulin resistance, dyslipidemia**	***CD36*** **7q21.11**	**Positional identification of the gene, combined with genome-wide gene expression studies;*Cd36*is deleted in the SHR strain; transgenic expression of*Cd36*in SHR ameliorates insulin resistance and lowers serum fatty acids; association of human*CD36*with T2DM; the gene also controls blood pressure: see above**	[30–32]
**Diabetes, type 2: T2DM (*Nidd/gk1*)**	Inppl1** **1q33** **166.90 Mb**	**T2DM**	***INPPL1*** **11q13.4**	**Positional identification of the gene, mutated in the Goto-Kakizaki diabetic rat (and the insulin-resistant SHR); mutations were then found in human diabetic patients**	[452]
Diabetes, type 2: T2DM (diet-induced)	Ndufa4* 4, 38.23 Mb	–	*–*	Positional identification of the gene, which shows a 61 bp deletion, unique to the Cohen diabetic rat; this mutation adversely affects mitochondrial function and promotes diet-induced diabetes	[453]
Diabetes, type 2: T2DM (fat mass and insulin resistance)	*Pparg*^ENU^ 4, 147.27 Mb	Lipodystrophy and insulin resistance	*PPARG* 3p25.2	The heterozygous F344 missense mutant shows reduced fat mass with adipocyte hypertrophy and insulin resistance (the homozygous mutant is lethal)	[454]
Diabetes, type 2: T2DM (*Dmo1*)	Prlhr** 1, 289.10 Mb	Blood pressure	*PRLHR* 10q26.13	Positional identification of the gene; point mutation at translation initiation codon in the OLETF rat; the mutation causes hyperphagia	[455]
Diabetes, type 2: T2DM	*Tbc1d4*^T^ 15, 85.93 Mb	Increased T2DM risk	*TBC1D4* 13q22.2	The Wistar KO mutant shows glucose intolerance and insulin resistance	[456]
Diabetes, type 2: T2DM (beta cell lipotoxicity)	*Tlr4*^T^ 5, 82.59 Mb	–	*–*	The SD KO mutant shows delayed damage induced by high-fat diet, improved beta-cell function, decreased pancreatic inflammatory infiltration and apoptosis; see also below, Inflammation	[457]
**Diabetes, type 2: T2DM**	Tpcn2*** **1, 218.42 Mb**	**Fasting insulin**	***TPCN2*** **11q13.3**	**QTL was detected in a HS; differential expression of*Tpcn2*; nonsynonymous coding variant as well as other SNPs were associated with fasting glucose;*TPCN2*was associated with fasting insulin in humans**	[458]
Diabetes, type 2: T2DM (Diabetic kidney disease)	*Trpc6*^T^ 8, 6.81 Mb	Familial focal segmental glomerulosclerosis	*TRPC6* 11q22.1	The results indicate that TRPC6 channel inhibition (in the SS rat background) has partial renoprotective effects in diabetic rats	[459]
Encephalo-myelitis (EAE)	*Cd8a*^ENU^ 4, 163.99 Mb	–	*–*	The KO Lewis mutant is protected from EAE	[460]
EAE	Clec4d** 4, 156.25 Mb, and Clec4e** 4, 156.27 Mb	Multiple sclerosis	*–*	Positional identification of the genes; rat strains expressing lower levels of *Clec4d* and *Clec4e* on myeloid cells exhibit a drastic reduction in EAE incidence; the CLEC4D/CLEC4E signaling pathway is upregulated in peripheral blood mononuclear cells from MS patients	[461]
EAE	Dlk1** 6, 142.74 Mb	IDDM (depending of parental origin)	*DLK1* 14q32	Parent-of-origin dependent QTL; the paternal PVG risk allele predisposes to low *Dlk1* expression	[462]
EAE: *Eae1*	Btnl2* 20p12, 6.22 MB and RT1-Db1* 20p12, 6.17 Mb	Multiple sclerosis	*HLA-DRB1* 6p21.3	Positional identification: the two genes in the MHC class II locus were identified in a HS and are the best candidate variants, amongst 3 candidate genes	[350]
**EAE:*Eae30***	Rgma* **1, 134.70 Mb**	**Multiple sclerosis**	***RGMA*** **15q26.1**	**Positional identification of the rat gene but polymorphisms of*Rgma*were not sought; it is thus a suggestive causal gene; however this result led to the discovery that a SNP in*RGMA*is associated with multiple sclerosis in humans**	[463]
**EAE:*Eae4***	Vav1** **9q12, 8.6 Mb**	**Multiple sclerosis**	***VAV1*** **19p13.2**	**Positional identification of the gene: one SNP in rat exon 1 correlates with EAE susceptibility and high TNF; in humans, association found between*VAV1*haplotype (high expression) and multiple sclerosis; the gene also regulates arthritis (see above)**	[341, 464]
**EAE:*Eae31; Pia32***	Il21r* **1, 197.00 Mb**	**Multiple sclerosis**	***IL21R*** **16p12.1**	**Positional identification of the rat gene but polymorphisms of*Il21r*were not sought; however this result led to the discovery that SNP’s in*IL21R*are associated with multiple sclerosis in humans**	[463]
**EAE:*Eae29; Pia8***	Il22ra2** **1, 15.09 Mb**	**Multiple sclerosis**	***IL22RA2*** **6q23.3**	**The susceptible strain DA carries a unique variant of the gene, which is differently expressed; a SNP in*IL22RA2*was associated with multiple sclerosis in humans**	[330, 465]
Experimental autoimmune neuritis: *Ean6*	Ncf1* 12, 25.50 Mb	Guillain-Barré syndrome	*–*	Positional identification of the gene, a suggestive causal gene: no polymorphism between strains was sought but functional studies support the role of *Ncf1* (the gene also controls EAE and PIA: see above)	[466]
Epilepsy (idiopathic, generalized; GAERS)	Cacna1h** 10, 14.73 Mb	Absence epilepsy	*CACNA1H* 16p13.3	Direct sequencing of the gene showed a mutation in the Genetic Absence Epilepsy Rats from Strasbourg (and not in non-epileptic strains); in an F2 cross, the phenotype segregates with the mutation	[467]
Epilepsy, tremor	Hcn1**^, T^ 2, 50.10 Mb	Infantile epileptic encephalopathy	*HCN1* 5p12	Positional identification of the gene; a typical example of epistasis: rats (TRM/Kyo) possessing a large deletion (*tm*) on chromosome 10 (240 Kb; 13 genes) exhibit tremor if they also possess the allele *Hcn1*^*A354V*^; when this allele is replaced by *Hcn1*^*V35A*^ tremor is absent (TRMR rats); subsequently, an F344 KO mutant was generated and showed susceptibility to induced seizure	[468, 469]
Glomerulonephritis *(Crgn8)*	Cp** 2, 104.74 Mb	–	*–*	Positional identification of the gene in combination with genome-wide eQTL mapping and functional tests; ceruloplasmin is overexpressed in WKY macrophages	[470]
**Glomerulonephritis*(Crgn1)***	Fcgr3-rs** **13, 89.38 Mb**	**Glomerulonephritis in systemic lupus** **erythematosus**	***FCGR3B*** **1q23.3**	**Positional identification of the loss of an*Fcgr3*paralog as a determinant of glomerulonephritis in WKY rats; expressing the gene in primary WKY macrophages results in low levels of phagocytosis; in humans, association found between low copy number of*FCGR3B*and lupus nephritis**	[471, 472]
Glomerulonephritis *(Crgn2)*	Jund** 16, 20.48 Mb	–	–	Localization of a QTL and genome-wide gene expression studies associated upregulation of *Jund* (due to a SNP in the promoter region) with glomerulonephritis; *Jund* KO in primary macrophages led to reduced macrophage activity	[473]
Glomerulonephritis	Kcnn4** 1, 81.22 Mb	–	–	Genome-wide eQTL mapping in macrophages from a segregating population led to the identification of *Kcnn4* as a key regulator of macrophage multinucleation and inflammatory diseases; *Kcnn4* is trans-regulated by *Trem2*	[474]
Glucose homeostasis	*Tbc1d1*^T^ 14, 45.60 Mb	CAKUT	*TBC1D1* 4p14	The SD KO mutant shows impaired contraction-induced sarcolemmal glucose transporter 4 redistribution, impaired glucose-tolerance and reduced pancreatic beta-cell mass	[475–477]
Heart failure	Ephx2** 15, 42.76 Mb	–	–	Localization of a QTL and genome-wide gene expression studies associated upregulation of *Ephx2* (due to a sequence variation in the promoter region) with heart failure susceptibility	[478]
Herpes simplex encephalitis susceptibility: *Hse1*	Calcr* 4q13, 28.53 Mb	–	–	Differences in expression level of *Calcr* mRNA and in protein localization between the susceptible (DA) and resistant (PVG) strains	[479]
Hippocampus function	*Trpm4*^T^ 1, 101.29 Mb	–	–	The SD KO mutant shows a distinct deficit in spatial working and spatial memory as well as changes in various target regions of the right dorsal hippocampus upon stimulation of Schaffer collaterals	[480, 481]
**Inflammation: Irf7-driven inflammatory network**	Gpr183** **15q15,** **108.36 Mb**	**IDDM**	***GPR183*** **13q32**	**Gene expression analyses and QTL mapping done in the rat; the results were translated to humans, identifying*GPR183*(=*EBI2)*as a type 1 diabetes susceptibility gene**	[482]
Inflammation: TNF induction	*Tlr4*^T^ 5, 86.69 Mb	–	–	The Wistar KO rat shows markedly reduced TNF induction upon liposaccharide challenge; see also above, Diabetes, type 2	[483]
Insulin resistance	Pparg**			See above, Diabetes type2, Fat mass	
Macrophage development	*Csf1r*^T^ 18, 56.41 Mb	ALSP	*CSF1R* 5q32	The DA KO mutant shows multiple abnormalities: loss of macrophages in several organs, osteopetrosis, infertility, lack of tooth eruption, loss of visceral fat, absence of microglia; see above, Mongenetic traits, *toothless* for mutation in *Csf1*	[60]
Macrophage function	*Cyp2j4*^T^ 5, 119.55 Mb	–	*–*	The WKY KO mutant macrophages show a profibrotic transcriptome; the macrophage epoxygenase could thus play a role in fibrotic disorders with inflammatory component; see also below, Metabolic syndrome	[484]
Metabolic syndrome (*Niddm30*)	*Camk2n1*^T^ 5, 156.88 Mb	Elevated risk of T2DM and coronary heart disease	*CAMK2N1* 1p36.12	The gene was a solid candidate gene for metabolic syndrome (blood pressure, diabetes, left ventricule weight); the SHR KO rat shows reduced cardiorenal Camk2 activity, lower blood pressure, lower left ventricular mass, decreased visceral fat mass and increased insulin sensitivity	[485]
**Metabolic syndrome**	***Cfb***^**T**^ **20p12,** **4.54 Mb**	**NIDDM and components of metabolic syndrome**	***CFB*** **6p21.33**	**The SHR KO rat shows improved glucose tolerance and adipose distribution, lower blood pressure, and reduced left ventricular mass; several human SNPs in*CFB*were associated with cardiometabolic traits**	[434]
Metabolic syndrome	*Cyp2j4*^T^ 5, 119.55 Mb	–	–	The WKY KO mutant shows adipocyte hypertrophy and weight gain; under “cafetaria diet”, it shows hepatic lipid accumulation, dysregulated gluconeogenesis and increased triglyceride levels; see also above, Macrophage function	[486]
Metabolic syndrome	Folh1** 1, 150.32 Mb	–	–	Positional identification of the gene; the SHR allele shows 2 missense mutations; an SHR congenic line harboring the BN *Folh1* allele shows decreased glucose and insulin concentrations	[487]
Metabolic syndrome	Folr1*** 1, 166.93 Mb	–	–	Positional identification of the gene, the promoter of which is mutated in the SHR; transgenic rescue experiments ameliorate most of the metabolic disturbances, probably linked to folate deficiency and hypercysteinemia	[488]
Metabolic syndrome	Gja8**2, 199.05 Mb	–	–	The *Gja8* mutation present in the SHR-Dca strain causes dominant cataract (see above, Monogenic traits); in the heterozygous form this mutation results in increased concentration of triacyl-glycerols, decrease of cholesterol and elevation of inflammatory cytokines	[489]
Metabolic syndrome	*Mt-Nd2, Mt-Nd4, Mt-Nd5*	–	–	The conplastic rat SHR-mt^LEW^ only differs from SHR in the sequence of these 3 mitochondrial genes and exhibits increased serum fatty acid levels and resistance to insulin stimulated incorporation of glucose into adipose tissue lipids	[490]
Metabolic syndrome	Wars2*** 2q34, 201.17 Mb	Cardio-metabolic phenotypes	*WARS2* 1p12	Positional identification of the gene; the SHR allele is mutated (and causes reduced angiogenesis – see above); transgenic SHR-*Wars2* rats exhibit increased glucose oxidation and incorporation into brown adipose tissue, as well as lower adiposity	[491]
Metabolic syndrome	*Zbtb16*^T^ 8, 51.57 Mb	–	*–*	The heterozygous SHR KO rat exhibits lower serum and triglycerides and cholesterol as well as increased sensitivity to adipose and muscle tissue to insulin action	[407]
Metabolic syndrome: obesity	Aqp11** 1, 162.70 Mb	–	*–*	Positional identification of the gene in combination with expression QTL mapping; the LH rat allele is mutated in the 3′ UTR and the 5′ upstream region; downregulation of *Aqp11* is associated with obesity in the LH rat; aquaporins are now considered to be involved in adipose tissue homeostasis	[492]
Metabolism	*Apoa4*^T^ 8q23, 50.54 Mb	–	–	The SD KO mutant shows improved glucose tolerance and altered expression of genes expressed in the liver, with enhanced glycolysis, attenuated gluconeogenesis and elevated de novo lipogenesis	[493]
Metabolism	*Esr1*^T^ 1q12, 41.19 Mb	–	–	The male SD KO liver shows altered expression of genes involved in carbohydrate and lipid metabolism; see also above, Monogenic traits, Infertility	[494]
Metabolism	*Pmch*^*ENU*^ 7, 28.65 Mb	–	–	The Wistar KO mutant is lean, hypophagic, osteoporotic and has a low adipose mass due to a lower adipocyte cell size	[495, 496]
Metabolism (steroid synthesis)	*Tspo*^T^ 7, 124.46 Mb	Anxiety-related disorders	*TSPO*	The SD KO mutant displays impaired ACTH-induced steroid production and reduced circulating testosterone levels; in humans a rare *TSPO* allele is associated with a reduced plasma cortisol rate of formation	[497]
Neuromyelitis optica spectrum disorders	*Cd59*^T^ 3, 94.01 Mb	–	–	The SD KO mutant shows no overt phenotype, except for mild hemolysis; however upon intracerebral administration of autoantibodies against astrocyte aquaporin 4, it shows marked neuromyelitis optica pathology including inflammation and demyelination	[498]
Non-alcoholic fatty liver disease	*Pten*^T^ 1, 251.42 Mb	–	–	This study reports the somatic inactivation of *Pten* in the liver (by injection of an inactivating plsmid); the treated SD rats showed increased body weight and triglyceride level, with increased lipid accumulation in the liver	[499]
Oxidative stress- mediated cell death	*Keap1*^T^ 8, 22.25 Mb	–	–	Grafting of mesenchymal stem cells is hampered by oxidative stress-mediated cell death; *Keap1* was knocked- down in adipose-derived mesenchymal stem cells leading to the activation of NFE2L2 and lower oxidative stress	[500]
Pain	*Scn9a*^T e^ 3, 52.58 Mb	–	–	The SD KO ^e^ rat does not exhibit nociceptive pain responses in hot plate nor neuropathic pain responses following spinal nerve ligation; inhibition of SCN9A in humans may thus reduce pain in neuropathic conditions	[501]
Pain	*Trpv1*^T^ 10, 59.80 Mb	–	–	Neuroimaging experiments of SD KO and WT rats showed that capsaicin-induced pain activates neuronal circuitries involved in pain but also in emotion and memory in a TRPV1-dependent manner; this channel was shown to be dispensable for hypernatremia-induced vasopressin secretion	[502, 503]
Pain (visceral nociception)	*Trpc4*^T^ 2, 143.43 Mb	–	–	The F344 KO rat is tolerant to noxious chemical stimuli applied to the colon (the gene is also involved in Blood pressure control -PAH- and Behavior, drug addiction: see above)	[504]
Pain processing	*Ano3*^T^ 3, 108.44 Mb	–	–	The F344 KO rat shows increased neuronal activity and increased thermal and mechanical sensitivity	[505]
Proteinuria (*Pur1*)	Actr3**13, 46.81 Mb	–	–	Positional identification of the gene: sole gene mutated in the *Pur1* interval of the BUF/Mna rat (a model of glomerulosclerosis)	[506]
Proteinuria	*Agtrap*^T^ 5, 168.55 Mb	Renal function	*AGTRAP-PLOD1* locus; 1p36	The SS KO rat shows decreased urinary protein excretion; the human locus had been identified in GWAS	[39]
Proteinuria	*Clcn6*^T^ 5, 168.47 Mb	Renal function	*AGTRAP-PLOD1* locus; 1p36	The SS KO rat shows decreased urinary protein excretion; the human locus had been identified in GWAS	[39]
Proteinuria	*Mthfr*^T^ 5, 168.50 Mb	Renal function	*AGTRAP-PLOD1* locus; 1p36	The SS KO rat shows increased urinary protein excretion; the human locus had been identified in GWAS and *MTHFR* could be linked to blood pressure and renal phenotype	[39]
Proteinuria	*Plod1*^T^ 5, 168.38 Mb	Renal function	*AGTRAP-PLOD1* locus; 1p36	The SS KO rat shows increased urinary protein excretion; the human locus had been identified in GWAS	[39]
Proteinuria (*Rf2*)	Rab38***^,T^ 1, 152.07 Mb	–	–	Natural KO in FHH; transgenesis in FHH and targeted KO in a FHH.BN congenic demonstrated the role of *Rab38* in protein excretion	[507]
Proteinuria and kidney damage	Add3*** 1q55, 273.85 Mb	–	*–*	Positional identification and sequencing of the FHH gene revealed a deleterious mutation; knockout and transgenesis experiments confirmed the causal role of the mutation	[508, 509]
Proteinuria and kidney damage (*Rf4*)	Shroom3** 14, 16.62 Mb	Renal function	*SHROOM3* (GWAS) 4q21.1	Congenic mapping and sequence analysis in rats suggested that *Shroom3* was a strong positional candidate gene; variants disrupting the actin-binding domain of *SHROOM3* may cause podocyte effacement and impairment of the glomerular filtration barrier in zebrafish	[510]
Proteinuria and kidney damage	*Tgfb*^T^ 1, 83.74 Mb	–	–	Heterozygous KO of *Tgfb* protects the SS rat against high salt-induced renal injury	[511]
Proteinuria and kidney damage	Tmem63c* 6, 111.04 Mb	–	–	Positional identification of the gene, which shows differential glomerular expression; the susceptible strain (MWF) also shows a nephron deficit; patients with focal segmental glomerulosclerosis exhibit loss of glomerular *TMEM63C* expression	[512]
**Proteinuria and kidney damage (*Pur7*?)**	Arhgef11** **2, 206.39 Mb**	**Glomerular filtration rate**	**1q21**	**Positional identification of the gene; allelic variants are differentially expressed in SS, SHR and congenic rats**	[513]
**Proteinuria and kidney disease (*Rf1*)**	Sorcs1**^,T^ **1, 277.40 Mb**	**Kidney disease**	***SORCS1*** **10q23-q25**	**The*Rf1*interval was narrowed down to a single gene,*Sorcs1*, which only shows polymorphisms in non-coding regions;*Sorcs1*KO in the consomic FHH-1**^**BN**^**causes increased proteinuria and impairment of albumin transport; in humans, association was found between*SORCS1*and kidney disease**	[514]
QT-interval	Rffl-lnc1***			See above, Blood pressure and QT-interval	[33]
Renal injury	*Nr4a1*^T^ 7, 142.90 Mb	**–**	–	The FHH KO rat shows early onset of kidney injury and progressive decline in kidney function resulting from macrophage-mediated enhanced inflammatory processes; the gene is also involved in dyskinesia (see above, Monogenic traits, Parkinson disease model) and in Behavior, Stress response (see above)	[515]
Renal injury	*Serpinc1*^T^ 13, 78.81 Mb	**–**	–	Patients with low SERPINC1 activities present a higher risk of developing AKI after cardiac surgery; the heterozygous congenic SS.BN KO rat shows increased renal injury after renal ischemia/reperfusion	[516]
Renal injury	Stim1*** 1, 167.37 Mb	Autoimmunity	*STIM1* 11p15.4	Genome sequencing revealed a deletion affecting the *Stim1* gene is the SHR-A3 which develops renal injury; a congenic line with a functional *Stim1* gene shows an improved lymphocyte function and a strong reduction in renal injury and glomerular immunoglobulin deposition; this pleiotropic genes also controls Diabetes insipidus (see above, Monogenic traits), Behavior (stress response, see above, Polygenic traits) and Stroke (see below)	[517]
Rheumatoid factor production	Igl** 11, 83.93 Mb	**–**	**–**	Analysis of congenic and advanced intercrossed rats showed that the *Igl* locus controls rheumatoid factor production and allergic bronchitis	[518]
Spermatogenesis	*Tp53*^T^ 10q24, 56.19 Mb	Li-Fraumeni syndrome	*TP53* 17p13.1	The SD KO mutant shows testicular atrophy, spontaneous spermatocyte death and germ cell depletion; *Tp53* also controls Cancer development: see above, Monogenic traits	[519]
Stroke	Igh* 6, ~ 138 Mb	–	*–*	Congenic substitution of the SHRSP *Igh* locus with the corresponding haplotype from SHR (stroke-resistant) reduced cerebrovascular disease, as well as the serum levels of autoantibodies to key cerebrovascular stress proteins	[520]
**Stroke*(Str1)***	Ndufc2*^,T^ **1, 162.37 Mb**	**Stroke**	***NDUFC2*11q14.1**	**Positional identification of the gene and differential expression study:*Ndufc2*is down-regulated in SHRSP (no sequence difference between SHRSP and SHRSR); the heterozygous SHRSR KO rat shows stroke occurrence and renal abnormalities, similarly to the SHRSP rat; in humans, association was found between*NDUFC2*and stroke**	[35, 36]
**Stroke (*Str2*)**	Nppa** **5, 165.81 Mb**	**Stroke**	***NPPA*** **1p36.21**	**Positional identification of the gene; altered sequence and expression of*Nppa*in the SHRSP rat; in humans, association was found between*NPPA*and stroke**	[521, 522]
Stroke	Stim1*** 1, 167.37 Mb	Autoimmunity	*STIM1* 11p15.4	Genome sequencing revealed a deletion affecting the *Stim1* gene is a stroke-prone subline (SHR-A3); a congenic line with a functional *Stim1* gene shows a strong reduction in stroke susceptibility; *Stim1* deficiency results in the generation of auto-antibodies; this pleiotropic genes also controls Diabetes insipidus (see above, Monogenic traits), Behavior (stress response) and Renal injury (see above, Polygenic traits)	[523]
Stroke: neuronal apoptosis	*Klf5*^T^ 15, 83.70 Mb	–	*–*	The SD KO rat with induced ischemic stroke shows reduction of infarct size and of apoptotic neuronal loss; KLF5 stimulates stroke-induced neuronal apoptosis	[524]
Stroke: neuronal apoptosis	*Mir195*^T^ 10, 56.84 Mb	–	*–*	The SD KO rat with induced ischemic stroke shows increased infarct size and apoptotic neuronal loss; Mir195 down-regulates *Klf5* expression	[524]
T-cell differentiation	*Pon1*^T^ 4, 30.25 Mb	–	*–*	The SD KO rat shows a decrease in CD4^+^, CD8^+^ and double-positive T-cells; PON1 prevents excessive apoptosis by inhibiting activation of the p38 signaling pathway	[525]
T-cell differentiation	Tap2** 20p12, 3.99 Mb + RT1-A** 20p12,? Mb	–	*–*	Positional identification of *Tap2* and *RT1-A*, which interact with one another and control CD4:CD8 ratio and MHC class expression	[526]
Toxicity	*Ahr*^T^ 6, 54.97 Mb	–	–	The SD KO mutant shows renal pathology and lack of responses to dioxin exposure (*Ahr* KO results in distinct phenotypes in mouse and rat)	[527]
Toxicity	*Nr1i2*^T^ 2, 65.02 Mb	–	*–*	An F344 KO mutant does not show the increase in NADPH-cytochrome P450 oxidoreductase protein and activity upon dexamethasone treatment; on the other hand, unlike wild-type rats, the SD KO rat fed diet containing pregnenolone-16alpha-carbonitrile (a non-genotoxic carcinogen) does not show increased thyroid gland weight	[528, 529]
Toxicity (liver)	*Nr1i3*^T^ 13, 89.59 Mb	–	*–*	Unlike wild-type rats, the SD KO rat fed diet containing sodium phenobarbital (a non-genotoxic carcinogen) does not show increased liver weight, hepatocyte replicative DNA synthesis and induction of cytochrome P450 enzymes	[529]
Vascular function	*Mc4r*^*ENU*^ 18, 62.61 Mb	Obesity	*MC4R*18q22	The MSH6 KO rat is obese (see above) and shows bradycardia and increased sympathetic tone to the vasculature	[530]
Vascular function	*Nfe2l2*^T^ 3, 623.50 Mb	–	–	The SD KO rat shows abnormalities in endothelium-dependent vasodilation and in microvessel density (*Nfe2l2* also controls aflatoxin B1 toxicity: see above)	[531]
Vascular function (vasodilation)	*Sod3*^*ENU*^ 14, 60.96 Mb	–	–	Missense mutation in the SS rat with deleterious effects on aortic vascular reactivity, but protective effects in mesenteric arteries; see also above, Chronic kidney disease	[532]
Vascular tone and nephropathy	*Shc1*^T^ 2, 188.75 Mb	–	–	The SS rat overexpresses *Shc1*, a feature linked to hypertension-induced increased renal damage; *Shc1* KO restores renal microvascular responses and mitigates glomerular damage in the SS rat	[533]

^a^ In forward genetic studies, the role of the causative genes is considered proven when complementation, mutation recovery, gene disruption or transgenesis was performed successfully (***); when these tests are lacking, the role of the gene can be either solid (**) (polymorphisms analysed in several contrasting strains, genetic linkage in a cross, or translation to genetic association in the human), or suggestive only (*) (for instance, polymorphism analysed in 2 contrasting strains only). Genes inactivated by ENU-driven target-selected mutagenesis are labeleled as ^ENU^. Targeted mutations (in general, KO rats) are labelled as ^T^

^b^ The human gene is indicated only when it has been implicated in the trait or diseases analysed in the rat

^c^ The gene positions are based on the data available at the NCBI (www.ncbi.nlm.nih.gov/), except those of the *Lta-Ncr3* region, derived from [338]; in the case of the rat, the cytogenetic position is indicated only when it was determined by in situ hybridization

^d^ The genomic scan of replicated high- and low-alcohol-drinking lines revealed signature of selection (excessive differentiation in the genomic architecture between lines) in 930 genes [320]; in the above table, only those genes residing in previously identified QTLs are quoted.

^e^ This mutant is in fact a knock-in mutant carrying a human insertion that, unexpectedly, was shown to be spliced out upon transcription, resulting in the generation of a premature stop codon and thus in a loss-of-function allele (except in the olfactory bulb)

Abbreviations used in the table:

1) Genes: *Abcb1a* ATP-binding cassette, sub-family B (MDR/TAP), member 1A (=*Mdr1a*, Multidrug resistance 1a/P-glycoprotein*)*, *Abcc2* ATP-binding cassette, sub-family C (CFTR/MRP), member 2 *(=Moat = Mrp2)*, *Abcc6* ATP binding cassette subfamily C member 6, *Abcc8* ATP binding cassette subfamily C member 8 (=*Sur1*, Sulfonylurea receptor 1), *Abcg2* ATP-binding cassette, sub-family G (WHITE), member 2 (Junior blood group) *(=Bcrp,* Breast cancer resistance protein), *Abcg5* ATP-binding cassette, sub-family G (WHITE), member 5, *ABCG8* ATP-binding cassette, sub-family G (WHITE), member 8, *Actr3* ARP3 actin-related protein 3 homolog (yeast), *Adamts16* Disintegrin and metallopeptidase with thrombospondin type 1 motif, 16, *Adcyap1r1* Adenylate cyclase activating polypeptide receptor type 1, *Add1* Adducing 1 (alpha), *Add3* Adducing 3 (gamma), *Agtr1a* Angiotensin II receptor, type 1a, *Adgrl3* Adhesion G protein-coupled receptor L3 (=*Lphn3*), *Adra2a* Adrenoceptor alpha 2A*, Ahr* Aryl hydrocarbon receptor, *Angptl8* Angiopoietin-like 8, *Anks6* Ankyrin repeat and sterile alpha motif domain containing 6 (= *Pkdr1, SamCystin)*, *Ano3* Anoctamin 3, calcium activated chloride channel (=*Tmem16c*), *Apc* Adenomatous polyposis coli, *Aplec* Antigen-presenting lectin-like receptor gene complex (=*Dcir3*), *Apoa4* Apolipoprotein A4, *Apoe* Apolipoprotein E, *Aqp4* Aquaporin 4, *Aqp11* Aquaporin 11, *Ar* Androgen receptor, *Arntl* Aryl hydrocarbon receptor nuclear translocator-like (=*Bmal1*), *Ar* Androgen receptor, *Arhgef11* Rho guanine nucleotide exchange factor (GEF) 11, *Arsb* Arylsulfatase B, *Asip* Agouti signaling protein, *Aspa* Aspartoacylase, *Atm* Ataxia-telangiectasia mutated serine/threonine kinase, *Atp7b* ATPase, Cu++ transporting, beta polypeptide, *Atrn* Attractin, *Avp* Arginin vasopressin, *Bace1* Beta-secretase 1, *Bckdk* Branched chain ketoacid dehydrogenase kinase, *Bdnf* Brain-derived neurotrophic factor, *Bglap* Bone gamma-carboxyglutamate protein (=osteocalcin), *Brca2* BRCA2, DNA repair associated, *Bscl2* BSCL2 lipid droplet biogenesis associated, seipin, *CIIta* Class II, major histocompatibility complex, transactivator *(=Mhc2ta)*, *C3* Complement C3, *Cacna1a* Calcium channel voltage-dependent subunit alpha 1A, *Cacna1c* Calcium voltage-gated channel subunit alpha1 C, *Cacna1f* Calcium voltage-gated channel subunit alpha1 F, *Cacna1h* Calcium voltage-gated channel subunit alpha1 H, *Calcr* Calcitonin receptor, *Camk2* Calcium/calmodulin-dependent protein kinase II, *Camk2n1* Calcium/calmodulin-dependent protein kinase II inhibitor 1, *Cav3* Caveolin 3, *Cblb* Cbl proto-oncogene B, *Ccdc39* Coiled-coil containing domain 39, *Ccdc85c* Coiled-coil containing domain 85C, *Cckar* Cholecystokinin A receptor, *Cd8a* Cd8A molecule, *Cd36* CD36 molecule, fatty acid translocase, *Cd59* Cd59 molecule, *Cd247* CD247 molecule (CD3 zeta chain), *Cdh13* Cadherin 13, *Cdkn1b* Cyclin dependent kinase inhibitor 1B, *Cfb* complement factor B, *Cftr* Cystic fibrosis transmembrane conductance regulator, *Chrm3* Cholinergic receptor, muscarinic 3, *Cit* Citron rho-interacting serine/threonine kinase, *CLEC4A* C-type lectin domain family 4, member A *(=DCIR)*, *Clec4b* C-type lectin domain family 4, member B, *Clec4d* C-type lectin domain family 4 member D (=*Mcl*), *Clec4e* C-type lectin domain family 4 member E (=*Mincle*), *Cntnap2* Contactin associated protein like 2, *Cntrob* Centrobin, centrosomal BRCA2 interacting protein, *Cp* Ceruloplasmin, *Cplx1* Complexin 1, *Crb1* Crumbs cell polarity complex component 1, *Crebbp* CREB binding protein (=*Cbp*), *Crhr2* Corticotropin releasing hormone receptor 2, *Cryba1* Crystallin beta A1, *Crygd* Crystallin gamma D, *Csf1* Colony stimulating factor 1, *Csf1r* Colony stimulating factor 1 receptor, *Ctnnd2* Catenin (cadherin-associated protein), delta 2, *Ctns* Cystinosin, lysosomal cystin transporter, *Cyba* Cytochrome b-245 alpha chain, *Cyp2c11* Cytochrome P450, family 2, subfamily c, polypeptide 11, *Cyp2e1* Cytochrome P450, family 2, subfamily e, polypeptide 1, *Cyp2j4* Cytochrome P450, family 2, subfamily j, polypeptide 4 (human *CYP2J2* ortholog, epoxygenase), *Cyp3a1/2* Cytpchrome P450, family 3, subfamily a, polypeptide 1/2, *Cyp4f18* Cytochrome P450, family 4, subfamily f, polypeptide 18, *Cyp11b1* Cytochrome P450, family 11, subfamily b, polypeptide 1, *Cyp17a1* Cytochrome P450 family 17, subfamily a, polypeptide 1, *Cyp27b1* Cytochrome P450 family 27 subfamily B member 1, *Dao* D-amino-acid oxidase, *Ddah1* Dimethylarginine dimethylaminohydrolase 1, *Defb23*/*26*/*42* Defensin beta 23/26/42, *Depdc5* DEP domain containing 5, *Dhh* Desert hedgehog, *Dmd* Dystrophin, *Disc1* Disc1 scaffold protein, *Dnd1* DND microRNA-mediated repression inhibitor 1, *Dnmt1* DNA methyltransferase 1, *Dock8* Dedicator of cytokinesis 8, *Dopey1* Dopey family member 1, *Dpp4* Dipeptidyl peptidase 4, *Drd1* Dopamine receptor D1, *Dsg4* Desmoglein 4, *Dusp5* Dual specificity phosphatase 5, *Endog* endonuclease G, *Ephx2* Epoxide hydrolase, *Ercc6* ERCC excision repair 6, chromatin remodelling factor (=*Csb*: Cockayne syndrome B), *Esr1* Estrogen receptor 1, *Esr2* Estrogen receptor 2, *Edaradd* EDAR-associated death domain*, Ednrb* Endothelin receptor type B, *F8* Coagulation factor F8, *Fah* Fumarylacetoacetate hydrolase, *Fam129c* Family with sequence similarity 129, member C, *Fbxo10* F-box protein 10, *Fcgr1a* Fc fragment of IgG receptor Ia, *Fcgr2a* Fc fragment of IgG receptor IIa, *FCGR3B* Fc fragment of IgG receptor IIIb, *Fcgr3-rs* Fc fragment of IgG receptor III related sequence, *Fdft1* Farnesyl diphosphate farnesyltransferase1, *Fh* fumarate hydratase, *Fkbp5* FKBP prolyl isomerase 5, *Flcn* Folliculin (=*Bhd*, Birt-Hogg-Dube syndrome homolog), *Fmr1* Fragile X mental retardation 1, *Folh1* Folate hydrolase 1, *Folr1* Folate receptor 1, *Foxn1* Forkhead box N1, *Frem2* FRAS1 related extracellular matrix protein 2, *Frmpd1* FERM and PDZ domain containing 1, *Fry* Furry homolog (Drosophila), *Gdnf* Glial cell derived neurotrophic factor, *Gh* growth hormone, *Ghsr* Growth hormone secretagogue (ghrelin) receptor, *Gimap5* GTPase, IMAP family member 5 (=*Ian4/5*), *Git2* GIT ArfGAP 2, *Gja3* Gap junction protein, alpha 3, *Gja8* Gap junction protein, alpha 8 (=*Cox50*), *Gla* Galactosidase alpha, *Gnal* G protein subunit alpha L, *Golgb1* Golgin B1, *Gper1* G protein-coupled estrogen receptor 1, *Gpr183* G protein-coupled receptor 183 (*=Ebi2*), *Grin2a* Glutamate ionotropic receptor NMDA type subunit 2A, *Grm2* Glutamate metabotropic receptor 2 (=*mGlur2*), *Hcn1* Hyperpolarization activated cyclic nucleotide gated potassium channel 1, *Hip1* Huntington-interacting protein 1, *Hmx1* H6 family homeobox 1, *Hr* Hair growth associated, *Hsd11b2* Hydroxysteroid 11-beta dehydrogenase 2, *Htr7* 5-hydroxytryptamine (serotonin) receptor 7, adenylate cyclase-coupled, *Igh* Immunoglobulin heavy chain locus, *Igl* Immunoglobulin lambda chain complex, *Il1rl2* Interleukin 1 receptor like 2 *(=Il36r)*, *Il2rg* Interleukin 2 receptor, gamma, *Il21r* Interleukin 21 receptor, *Il22ra2* Interleukin 22 receptor, alpha 2, *Inppl1* Inositol polyphosphate phosphatase like 1, *Isca1* Iron-sulfur complex assembly 1, *Jund* JunD proto-oncogene, AP-1 transcription factor subunit, *Kcna1* Potassium voltage-gated channel, shaker-related subfamily, member 1, *Kng2* Kininogen 2, *Kcnj1* Potassium voltage-gated channel subfamily J member 1 (=*Romk*), *Kcnj10* Potassium voltage-gated channel subfamily J member 10 (=*Kir4.1*), *Kcnj16* Potassium voltage-gated channel subfamily J member 16, *Kncq1* Potassium voltage-gated channel, KQT-like subfamily, member 1, *Kcnk3* Potassium two pore domain channel subfamily K member 3, *Kcnn2* Potassium calcium-activated channel subfamily N member 2, *Kcnn4* Potassium calcium-activated channel subfamily N member 4, *Keap1* Kelch like ECH associated protein 1, *Kiss1* KISS-1 metastasis-suppressor (kisspeptin), *Kit* v-kit Hardy-Zuckerman 4 feline sarcoma viral oncogene homolog, *Klf5* Kruppel-like factor 5, *Krt@* Cytokeratin gene locus (type II), *Krt71* Keratin 71, *L1cam* L1 cell adhesion molecule, *Lamp2* Lysosomal associated membrane protein 2, *Ldlr* Low density lipoprotein receptor, *Lep* Leptin, *Lepr* Leptin receptor, *Lgi1* Leucine rich glioma inactivated 1, *Lipa* Lipase A, lysosomal acid, cholesterol esterase, *Lmx1a* LIM homeobox transcription factor 1, alpha, *Lpar1* Lysophosphatidic acid receptor 1, *Lpin1* Lipin 1 (phosphatidate phosphatase), *Lrp5* LDL receptor related protein 5, *Lrrk2* Leucine-rich repeat kinase 2, *Lss* Lanosterol synthase (2,3-oxidosqualene-lanosterol cyclase), *Lta* Lymphotoxin alpha, *Ltb* Lymphotoxin beta, *Lst1* Leukocyte-specific transcript 1, *Lyst* Lysosomal trafficking regulator, *Mas1* MAS1 proto-oncogen, G-protein-coupled recptor, *Mbd2* Methyl CpG binding domain binding protein 2, *Mbp* Myelin basic protein, *Mc4r* Melanocortin 4 receptor, *Mecp2* Methyl-CpG binding protein 2, *Mertk* MER proto-oncogene, tyrosine kinase, *Mip* Major intrinsic protein of lens fiber, *Mir31* Micro RNA 31, *Mir146b (5p)* Micro RNA 146b, *Mir195* Micro RNA 195, *Mkx* Mohawk homeobox, *Mrs2* MRS2 magnesium transporter, *Msh6* MutS homolog 6, *Mstn* Myostatin, *Mt-Nd2*, *Mt-Nd4*, *Mt-Nd5* Mitochondrial subunits Nd2, Nd4, Nd5 encoding the NAD dehydrogenase (complex I), *Muc1* Mucin 1, cellsurface associated, *Myh7b* Myosin heavy chain 7B, *Myo5a:*Myosin VA *Myo7a* Myosin VIIA, *Myo9b* Myosin IXB, *Myo15a* Myosin XVA, *Myl4 Myosin,* light chain 4, *Ncf1* Neutrophil cytosolic factor 1 (encodes the 47-kilodalton cytosolic subunit of neutrophil NADPH oxidase), *Ncf2* Neutrophil cytosolic factor 2 (=*p67phox;* 7-kilodalton cytosolic subunit of neutrophil NADPH oxidase), *NCF4* Neutrophil cytosolic factor 4, 40 kDa, *Ncr3* Natural cytotoxicity triggering receptor 3*, Ndufa4* NADH dehydrogenase 1 alpha subcomplex 4, *Ndufc2* NADH:ubiquinone oxidoreductase subunit C2, *Nek8* NIMA-related kinase 8, *Nfe2l2* Nuclear factor, erythroid 2 like 2 (*=Nrf2*), *Ngly1* N-glycanase 1, *Nlgn3* Neuroligin-3, *Nlrp1* NLR family, pyrin domain containing 1, *Nox4* NADPH oxidase 4, *Nppa* Natriuretic peptide A *(=Anp)*, *Nppb* Natriuretic peptide B (=*Bnp*), *Nppc* Natriuretic peptide C (=*Cnp*), *Npy* Neuropeptide Y, *Nr1i2* Nuclear receptor subfamily 1 group I member 2 (=*Pxr,* Pregnane X receptor), *Nr1i3* Nuclear receptor subfamily 1 group I member 3 (=*Car,* Constitutive androstane receptor), *Nr2f2* Nuclear receptor subfamily 2 group F member 2, *Nr3c1* Nuclear receptor subfamily 3 group C member 1 (=*Gr,* Glucocorticoid receptor), *Nrg1* Neuregulin 1, *Nur4a1* Nuclear receptor subfamily 4 group A member 1 (=*Nur77*), *Oca2* Oculocutaneous albinism II, *Ogdh* Oxoglutarate dehydrogenase, *Ogn* Osteoglycin, *Oprl1* Opioid related nociceptin receptor 1 (nociceptin/orphanin FQ receptor), *P2rx7* Purinergic receptor P2X7, *P2ry14* Purinergic receptor P2Y14, *Pappa1* Pappalysin 1, *Pappa2* Pappalysin 2, *Park7* Parkinson protein 7 (=*Dj1*), *Pax6* Paired box 6, *Pcdh15* Protocadherin 15, *Pclo* Piccolo presynaptic cytomatrix protein, *Pde3a* Phosphodiesterase 3A, *Pde6b* Phosphodiesterase 6B, *Phkg2* Phosphorylase kinase, gamma 2 (testis), *Pgls* 6-phosphogluconolactonase, *Phf24* PHD finger protein 24, *Pi15* peptidase inhibitor 15, *Pink1* Pten induced putative kinase, *Pkhd1* Polycystic kidney and hepatic disease 1 (autosomal recessive), *Plekha7* Pleckstrin homology domain containing family A member 7, *Plekhm1* Pleckstrin homology domain containing, family M (with RUN domain) member 1, *Plp1* Proteolipid protein 1, *Pmch* Pro-melanin-concentrating hormone, *Pon1* Paraoxonase 1, *Ppp4r3b* Protein phosphatase 4 regulatory subunit 3B (=*Smek2*), *Pparg* Peroxisome proliferator activated receptor gamma, *Prdm14* PR/SET domain 14, *Prdx2* Peroxiredoxin 2, *Prkdc* Protein kinase, DNA-activated, catalytic polypeptide, *Prkg2* Protein kinase, cGMP-dependent, type II, *Prkn* Parkin RBR E3 ubiquitin protein ligase (=*Park2*), *Prlhr* Prolactin releasing hormone receptor (=*Gpr10*), *Prss8* Protease, serine, 8, *Pten* Phosphatase and tensin homolog, *Ptprk* Protein tyrosine phosphatase, receptor type, K, *Rab38* RAB38, member RAS oncogene family, *Rag1* Recombination activating gene 1, *Rag2* Recombination activating gene 2, *Rarres2* Retinoic acid receptor responder 2 (=chemerin), *Rbm20* RNA binding motif protein 20, *Rffl* Ring finger and FYVE like domain containing E3 ubiquitin protein ligase (rififylin), *Rffl-lnc1 Rffl*-long non-coding RNA, *RT1-A* RT1 class I, locus *A*, *Rin1* Ras and Rab interactor 1, *RT1-Ba* RT1 class II, locus Ba, *RT1-Bb* RT1 class II, locus *Bb*, *Reln* Reelin, *Ren* Renin, *Resp18* Regulated endocrine-specific protein 18, *Rgma* Repulsive guidance molecule BMP co-receptor a, *Rnaset2* Ribonuclease T2, *Sbf1* SET binding factor 1, *Scn1a* Sodium channel, voltage-gated, type I, alpha subunit, *Scn9a* Sodium voltage-gated channel alpha subunit 9 (=Nav 1.7), *Serpinc1* Serpin family C member 1 (=antithrombin III), *Sh2b3* SH2B adaptor protein 3 *(=Lnk)*, *Shank2* SH3 and multiple ankyrin repeat domains 2, *Shank3* SH3 and multiple ankyrin repeat domains 3, *Shc1* SHC adaptor protein 1, *Shroom3* Shroom family member 3, *Slc6a3* Solute carrier family 6 member 3 (=DAT, dopamine transporter), *Slc6a4* Solute carrier family 6 member 4 (= SERT, serotonin transporter), *Slc11a2* Solute carrier family 11 (proton-coupled divalent metal ion transporter), member 2 (=*Nramp2*), *Slc22a18* Solute carrier family 22, member 18, *Slc39a12* Solute carrier family 39 member 12 (zinc transporter ZIP12), *Slco1b2* Solute carrier organic anion transporter family member 1B2, *SLCO1B3* Solute carrier organic anion transporter family member 1B3, *Snca* Synuclein alpha, *Sod3* Superoxide dismutase 3, extracellular, *Sorcs1* Sortilin-related VPS10 domain containing receptor 1, *Sp6* Sp6 transcription factor, *Spata22* Spermatogenesis associated 22, *Stim1* Stromal interaction molecule 1, *Sv2a* synaptic vesicle glycoprotein 2A, *Tap2* Transporter 2, ATP-binding cassette, sub-family B (MDR/TAP), *Tbc1d1* TBC1 domain family member 1, *Tbc1d4* TBC1 domain family member 4 (=*As160*), *Tbx6* T-box 6, *Tfr2* transferrin receptor 2, *Themis* Thymocyte selection associated, *Tg* Thyroglobulin, *Tlr4* Toll-like receptor 4, *Tmem63c* Transmembrane protein 63c, *Tmem67* Transmembrane protein 67 (=meckelin, *Mks3*), *Tp53* Tumor protein 53, *Tph2* Tryptophan hydroxylase 2, *Tpcn2* Two pore segment channel 2, *Trem2* Triggering receptor expressed on myeloid cells 2, *Trpa1* transient receptor potential cation channel, subfamily A, member 1, *Trpc4* Transient receptor potential cation channel, subfamily C, member 4, *Trpc6* Transient receptor potential cation channel subfamily C member 6, *Trpm4* Transient receptor potential cation channel subfamily M member 4, *Trpv1* Transient receptor potential cation channel subfamily V member 1, *Trpv3* Transient receptor potential cation channel, subfamily V, member 3, *Trpv4* Transient receptor potential cation channel subfamily V member 4, *Tsh* Thyroid stimulating hormone receptor, *Tspo* Translocator protein, *Tubb4a* Tubulin beta 4A class Iva, *Tyr* Tyrosinase, *Uba6* Ubiquitin-like modifier activating enzyme 6, *Ubd* Ubiquitin D (=*Fat10*), *Ube3a* Ubiquitin protein ligase E3A, *Ugt1a1* UDP glycosyltransferase 1 family, member A1, *Unc5c* unc-5 netrin receptor 5 (*=Unc5h3*), *Uox* Urate oxidase, uricase, *Vav1* Vav1 guanine nucleotide exchange factor, *Vdr* Vitamin D receptor, *Vkorc1* Vitamin K epoxide reductase complex, subunit 1, *Wars2* Tryptophanyl tRNA synthetase 2, mitochondrial, *Wfs1* Wolframin ER transmembrane glycoprotein, *Wwox* WW domain-containing oxidase, *Zbtb16* Zinc finger and BTB domain containing 16 (=*Plzf*)

2) Phenotypes and diseases: *ADLTE* Autosomal dominant lateral temporal lobe epilepsy, *ADPKD* Autosomal dominant polycystic kidney disease, *AKI* Acute kidney injury, *ALSP* Adult-onset leukoencephalopathy with axonal spheroid and pigmented glia, *AMD* Age-related macular degeneration, *ARPKD* Autosomal recessive polycystic kidney disease, *CAKUT* Congenital anomalies of the kidneys and the urinary tract, *CDFE* Cortical dysplasia-focal epilepsy, *CV* Cardiovascular, *DJS* Dubin-Johnson syndrome, *EA2* Episodic ataxia type 2, *EAE* Experimental autoimmune encephalomyelitis, *FHM1* Familial hemiplegic migraine type 1, *HNPCC* Hereditary non-polyposis colorectal cancer, *HPS* Hermansky-Pudlak syndrome, *HTNB* Hypertension with brachydactyly (autosomal dominant), *IBD* Inflammatory bowel disease, *IFAP* Ichthyosis follicularis, atrichia, and photophobia, *LVH* Left ventricular hypertrophy, *LVM* Left ventricular mass, *PAH* Pulmonary artery hypertension, *PCH3* Pontocerebellar Hypoplasia type 3, *PD* Parkinson disease, *PIA* Pristane-induced arthritis, *PKHD1* Polycystic kidney and hepatic disease 1, *RA* Rheumatoid arthritis, *RV* Right ventricular, *SAME* Syndrome of apparent mineralocorticoid excess, *SCA6* Autosomal dominant spino-cerebellar ataxia 6, *T1DM* Type 1 diabetes mellitus (Insulin-dependent diabetes mellitus), *T2DM* Type 2 diabetes mellitus (Non-insulin-dependent diabetes mellitus), *VKCFD2* Combined deficiency of vitamin K dependent clotting factors type 2, *(X-)SCID* (X-linked) severe combined immunodeficiency

3) Others: *ACTH* Adrenocorticotropic hormone, *CaMK* Ca^2+^/calmodulin-dependent protein kinase, *CNS* Central nervous system, *CRISPR-Cas* Clustered regularly interspaced short palindromic repeat, *ERE* estrogen-responsive-element, *ENU* N-ethyl-N-nitrosourea, *eQTL* Expression quantitative trait locus, *FHH* Fawn-hooded hypertensive, *GLP1* Glucagon-like peptide 1, *HDL* High density lipoproteins, *HPA* Hypothalamus-pituitary-adrenal, *HS* Heterogeneous stock, *Ig* Immunoglobulins, *IGF-1* Insulin-like growth factor-1, *KO* Knockout, *LDL* Low density lipoprotein, *LEW* Lewis, *LH* Lyon hypertensive, *LOH* Loss of heterozygosity, *mTORC1* mTOR complex 1 (*MTOR* = mechanistic target of rapamycin kinase), *MWF* Munich Wistar Frömter, *QTL* Quantitative trait locus, *SD* Sprague-Dawley, *SNP* Single nucleotide polymorphism, *SHR* Spontaneously hypertensive rat, *SHRSP* Spontaneously hypertensive rat, stroke prone, *SHRSR* Spontaneously hypertensive rat, stroke resistant, *SR* Dahl salt-resistant, *SS* Dahl salt-sensitive, *TNF* Tumor necrosis factor, *UTR* Untranslated transcribed region, *WT* Wild-type, *WKY* Wistar-Kyoto, *ZFN* Zinc finger nuclease

## Conclusions

This evaluation of progress in the identification of genes causing monogenic or polygenic rat diseases or related phenotypes yielded a list containing over 350 genes. In several instances the result obtained in the rat model was translated to the human, demonstrating that a considerable number of conserved genes have similar effects on biological traits in rats and humans, and thus providing one with a rich and useful resource of disease models (Table 1, bold entries). For instance, the *Inppl1* gene was first identified in the rat as a causative gene of type 2 diabetes and this discovery led to the identification of mutations in the homolog gene of diabetic patients [452, 534]. Similarly, a rat paralog of the *Fcgr3* gene (*Fcgr3-rs*) was identified as causing glomerulonephritis, and the result was promptly translated to the human: low copy number of *FCGR3B*, an orthologue of rat *Fcgr3*, was associated with glomerulonephritis in the autoimmune disease systemic lupus erythematosus [471].

Also, as mentioned above, even when the human gene causing a disease had been identified (without resorting to a rat model), mutated rat strains, in particular KO strains, were created to analyze the gene function and the disease pathogenesis, and, potentially, to develop new therapies.

This review illustrates the vigor of the rat biomedical research and its value for understanding the etiology of human diseases and for suggesting new therapies.

## Data Availability

Not applicable.

## Abbreviations

ENU: N-ethyl-N-nitrosourea
GWAS: Genome wide association study
KO: Knockout
NADPH: Nicotinamide adenine dinucleotide phosphate
QTL: Quantitative trait locus
RGD: Rat Genome Database
